# Intravital Multimodal Imaging of Human Cortical Organoid Transplantation in a Mouse Model of Chronic Stroke

**DOI:** 10.1002/advs.202515913

**Published:** 2026-07-14

**Authors:** Jinghui Wang, Guanda Qiao, Honglin Tan, Colleen Russel, Baixuan Yang, Mengyang Jacky Li, Kexin Wang, Jiadi Xu, Chengyan Chu, Miroslaw Janowski, Tian‐Ming Fu, Piotr Walczak, Yajie Liang

**Affiliations:** ^1^ Department of Diagnostic Radiology and Nuclear Medicine University of Maryland School of Medicine Baltimore MD USA; ^2^ Department of Electrical and Computer Engineering Princeton University Princeton NJ USA; ^3^ Department of Biomedical Engineering Johns Hopkins University Baltimore MD USA; ^4^ F.M. Kirby Research Center for Functional Brain Imaging Kennedy Krieger Research Institute Baltimore MD USA; ^5^ Russell H. Morgan Department of Radiology and Radiological Science Johns Hopkins University School of Medicine Baltimore MD USA; ^6^ Omenn Darling Bioengineering Institute Princeton University Princeton NJ USA

**Keywords:** brain organoids, MRI, multimodal intravital imaging, stroke, two‐photon microscopy

## Abstract

Chronic stroke poses enduring neurological deficits, while experimental strategies based on human induced pluripotent stem cell‐derived cortical organoids (COs) remain limited by the lack of tools for longitudinal in vivo graft monitoring. Here, we introduce the Multimodal Imaging Platform for Organoid Tracking (MIPOT), integrating MRI, bioluminescence imaging, surgical microscopy, and intravital two‐photon fluorescence microscopy to monitor CO transplantation in chronic stroke lesions in mice. MIPOT enabled confirmation of initial graft placement within cleaned infarct cavities, longitudinal assessment of viability‐associated bioluminescence signals, and high‐resolution visualization of graft‐derived cells and short‐term cellular dynamics. Bioluminescence imaging revealed a progressive decline in graft‐associated signals that stabilized at approximately 25% by two weeks, while endpoint histology confirmed limited persistence of human graft‐derived cells at later time points. Together, MIPOT provides a multimodal framework for monitoring organoid transplantation in the injured brain and supports future optimization of graft survival, maturation, and host–graft integration.

## Graphical abstract

A multimodal intravital imaging platform enables longitudinal tracking of human cortical organoids transplanted into chronic stroke lesions. By combining surgical microscopy, MRI, bioluminescence imaging, and two‐photon fluorescence microscopy, the platform captures graft placement, viability dynamics, and cellular‐scale morphology in vivo, offering a versatile framework for mechanistic studies and preclinical optimization of organoid‐based brain repair.

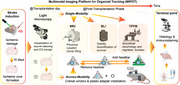

## 1 Introduction

Stroke is a leading cause of serious long‐term disability [1]. Despite notable advancements in acute stroke management, thrombolytic therapy and mechanical thrombectomy are still time‐sensitive (4.5 ‐6 h), and applicable to a limited number of stroke patients [2, 3]. As most return‐of‐function after stroke occurs early and recovery plateaus 3 to 6 months after stroke onset [4], chronic stroke, defined as 6 months to years after onset [5], has a very high chance of leading to permanent disability [6, 7]. Therefore, there is an unmet need for more effective therapeutic strategies that can address the complex, established neurological deficits of chronic stroke. Unfortunately, current therapeutic approaches have not yielded significant benefits for patients with chronic stroke, despite the immense efforts spent [8, 9].

Human induced pluripotent stem cell (hiPSC)‐derived brain organoids are self‐organizing, three‐dimensional (3D) structures that recapitulate key aspects of brain development, including the formation of region‐specific architectures such as the midbrain, hippocampus, and cerebellum [10, 11, 12]. Notably, the brain organoids show 3‐dimensional neural connectivity and brain functionality that recapitulate features of brain development and maturation [12, 13]. Compared to the transplantation of isolated neural stem or progenitor cells, brain organoid transplantation offers several advantages for neural repair. One major advantage is that they contain a structured microenvironment composed of diverse neural cell types—including progenitors, neurons, astrocytes, and oligodendrocytes [14, 15] that support each other and enhance graft survival following transplantation [16]. In addition, whereas transplantation of a single neural stem or progenitor cell type may be insufficient to regenerate all components of damaged brain tissue [17, 18], organoids provide a rich and varied cellular source capable of contributing to more comprehensive tissue reconstruction. Furthermore, brain organoids can be customized to mimic specific brain regions, allowing for the development of targeted therapies based on the anatomical and functional needs of the damaged area [16, 19, 20]. Together, these properties make brain organoids transplantation a promising approach for repairing or replacing damaged brain tissue for traumatic brain injury [21] and a range of neurodegenerative diseases [16, 19, 22].

Recent studies have explored the transplantation of hiPSC‐derived brain organoids for treating ischemic stroke in rodent models, demonstrating promising therapeutic effects [23, 24, 25]. However, two major challenges remain. First, the dynamic behavior of transplanted organoids is poorly understood due to the absence of intravital imaging tools. Most previous studies have relied on static histological analyses at terminal time points, providing only limited snapshots of graft positioning, survival, and cellular behavior. Second, it remains unclear whether brain organoid transplantation can be effectively applied to chronic stroke, as prior studies have primarily focused on the acute or subacute phases. To address these gaps, we developed a robust intravital multimodal imaging platform and applied it to study CO transplantation during the chronic phase of stroke in a mouse model. By integrating Magnetic Resonance Imaging (MRI), bioluminescence Imaging (BLI), bright field microscopy, and Two‐Photon Fluorescence Microscopy (TPFM), our multimodal imaging platform for organoid tracking (MIPOT) enables longitudinal, multi‐scale monitoring of graft location, viability, and morphological changes. We generated human COs via directed differentiation of hiPSCs and transplanted them into the infarct cavity following the surgical removal of the ischemic core at the chronic phase of stroke. Remarkably, the engrafted COs survived and exhibited obvious differentiation into neurons when they are transplanted in host hippocampus. Together, this study establishes a novel framework for evaluating brain organoid therapies in chronic stroke and underscores the value of multimodal imaging to track and quantify post‐transplantation graft status, ultimately advancing the clinical translation of organoid‐based treatment for chronic stroke.

## 2 Results

### 2.1 Multimodal Imaging Platform for Peri‐ and Post‐Transplantation Tracking of COs

**FIGURE 1:**
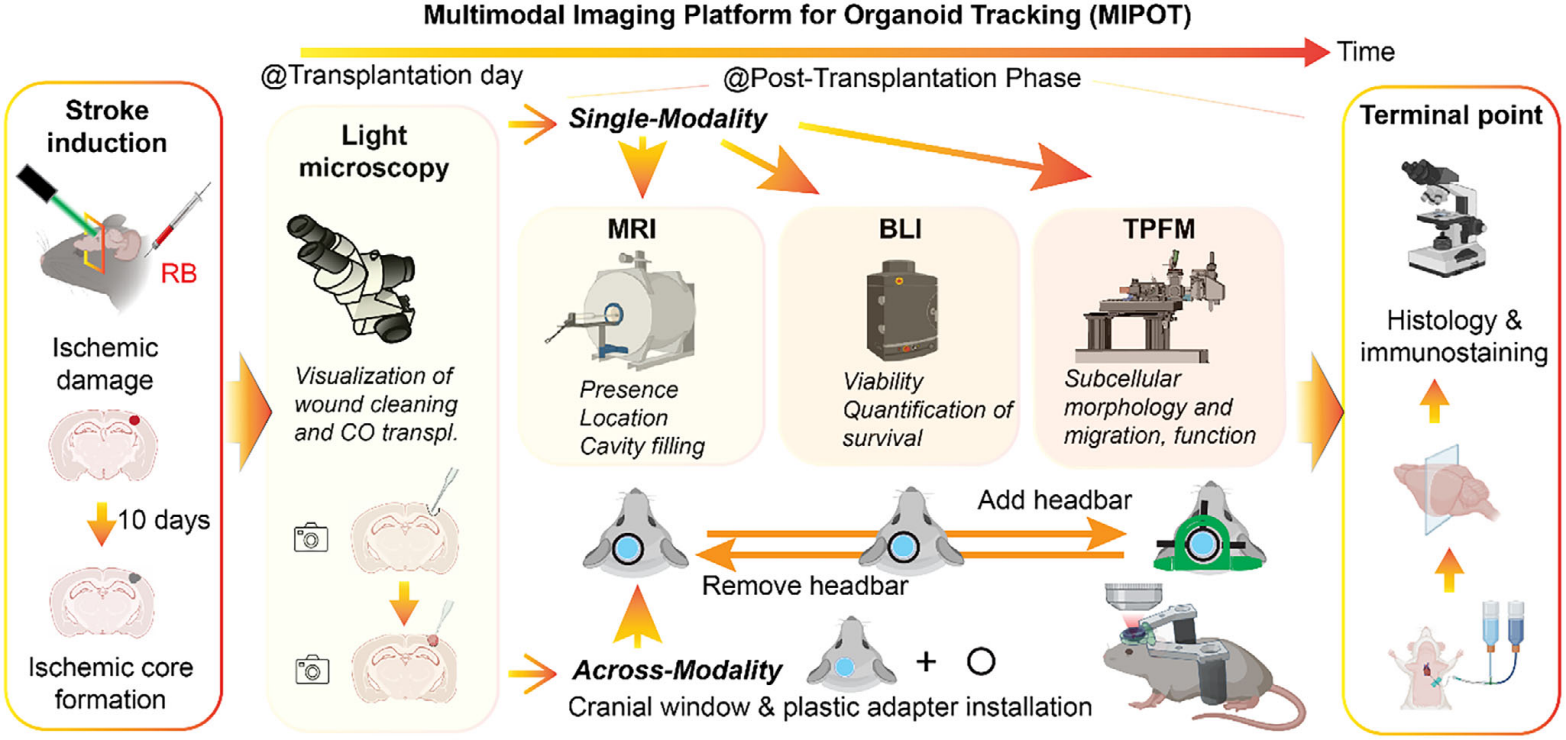
Workflow of the Multimodal Imaging Platform for Organoid Tracking (MIPOT). After Rose Bengal‐induced photothrombotic stroke and a 10‐day period of ischemic core formation, COs are transplanted under light microscopic guidance. During the post‐transplantation phase, MIPOT can be used in either a single‐modality configuration, in which MRI, BLI, and TPFM are performed separately, or an cross‐modality configuration, in which a cranial window and plastic adapter enable sequential imaging of the same animal across modalities. MRI is used to assess graft presence, location, and cavity filling; BLI is used to monitor graft viability and survival; and TPFM is used to visualize graft morphology at high resolution. Terminal histology and immunostaining provide ex vivo validation.

To enhance consistency, reliability, and quantitative assessment of organoid transplantation, we established the Multimodal Imaging Platform for Organoid Tracking (MIPOT). MIPOT integrates four complementary intravital imaging modalities (surgical microscopy, MRI, BLI, and TPFM) to monitor human iPSC‐derived COs transplanted into the chronically ischemic brain (Figure 1). Following photothrombotic stroke induction using Rose Bengal (RB), cortical infarcts are allowed to mature over a 10‐day period to model chronic stroke [26, 27, 28] before CO transplantation. MIPOT enables imaging across key phases of the transplantation workflow during peri‐transplantation period (Day −7 to Day +7 relative to organoid grafting, consistent with established clinical usage of ‘peri‐/periprocedural’ windows that span approximately one week before and after an index procedure [29, 30]). On the day of transplantation, light microscopy was used to visualize the ischemic core, guide wound cleaning, and monitor CO delivery into the lesion cavity. After transplantation, MIPOT can be implemented in two complementary modes. In the single‐modality mode, MRI, BLI, and TPFM are applied independently to characterize distinct aspects of the graft. MRI is used to assess graft presence, location, and cavity filling; BLI enables longitudinal measurement of graft viability and survival; and TPFM provides high‐resolution visualization of graft morphology and local cellular features. In the cross‐modality mode, implantation of a cranial window together with a plastic adapter enables sequential imaging of the same animal across modalities by allowing attachment or removal of the headbar as needed. At the terminal endpoint, histological and immunohistochemical analyses provide ex vivo validation of graft survival, phenotype, and host‐graft relationships. Together, MIPOT provides a flexible framework for dynamic evaluation of transplanted COs in vivo.

### 2.2 Generation of iPSC‐COs and their Transplantation for the Treatment of Chronic Stroke

**FIGURE 2:**
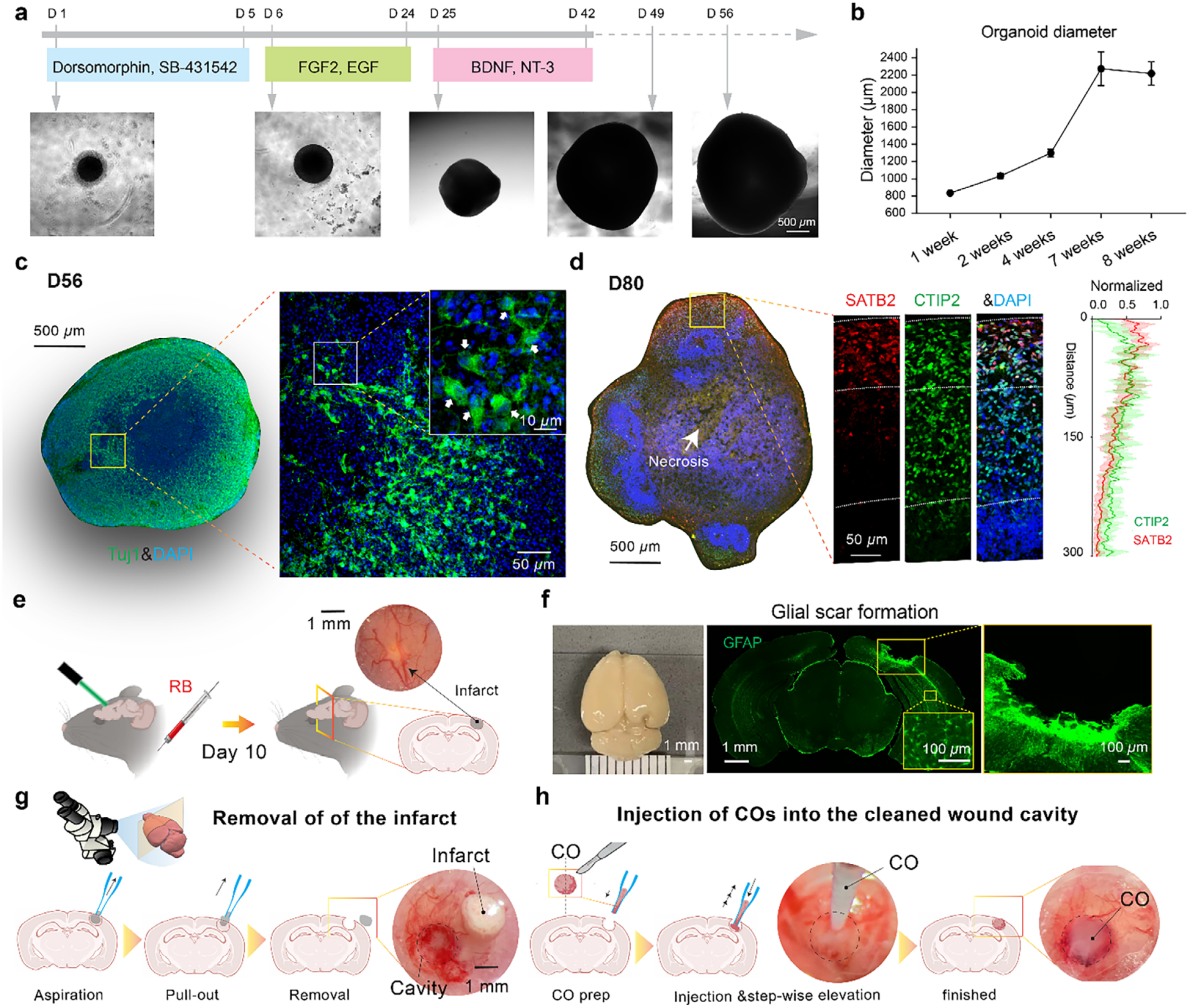
Generation of COs and the transplantation procedure for treatment of chronic stroke. (a) Timeline for CO differentiation. HiPSCs were differentiated into COs over 8 weeks using a step‐wise factor treatment. Representative brightfield images show organoid morphology at different time points. (b) Change of organoids diameter over time (n = 6 – 12 COs per timepoint, Mean and STD were plotted). (c) Immunohistochemistry of mature COs at D56 stained for Tuj1 (green, neuronal marker) and DAPI (blue, nuclei). The magnified inset (right) shows neuronal morphology and density, with white arrows indicating neuronal processes. (d) Spatial organization of cortical layers within D80 CO. Slices of D80 organoid (left panel) stained for SATB2 (purple, cortical layer II/IV marker), CTIP2 (green, cortical layer V/VI marker), and DAPI (blue, nuclei). The magnified inset (middle panel) illustrates the presence and spatial arrangement of distinct cortical neuron populations. Right, normalized fluorescence intensity profiles of SATB2 and CTIP2 along the longitudinal axis of the organoid, quantified from rectangular ROIs in ImageJ (n = 5 fields of view, each from a separate CO). (e) Schematic illustrating the induction of an ischemic infarct by injecting Rose Bengal (RB) and illuminating the brain with a 532 nm laser. The inset shows the visible infarct region in the mouse brain. Representative image of a mouse brain (left) and a brain slice showing the robust glial scar formation around the infarct site immunostained with GFAP antibody 10 days post onset of stroke. (g) Schematic depicting the aspiration procedure to create a clean wound cavity after infarct formation under direct visualization through the microsurgical microscope. The inset shows the resulting cavity and dissected infarct tissue from the mouse brain under a surgical microscope. (h) Schematic illustrating the surgical procedure for injecting COs into the prepared wound cavity in the mouse brain. The subsequent images show the organoid successfully placed within the cavity.

To generate COs enriched in cortical neuron subtypes, we adapted established protocols using dual SMAD inhibition and prolonged differentiation [31, 32]. (Figure 2a). Human iPSCs were first characterized by immunostaining of the featured TFs (Supplementary Figure S1a), then they were exposed to dual‐SMAD inhibitors (Dorsomorphin and SB‐431542) to induce neuroectodermal fate, followed by FGF2 and EGF to promote expansion of neural progenitors. Organoids maintained in this culture condition over 8 weeks exhibited characteristic changes in morphology, including size expansion and increased tissue density (Figure 2a,b and Supplementary Figure S1b,c). At day 56 (D56), immunostaining revealed robust neuronal differentiation as evidenced by widespread expression of βIII‐tubulin (Tuj1) across the organoid (Figure 2c). Higher magnification views showed processes of Tuj1+ cells (Figure 2c, arrows from insets), consistent with progressive neuronal differentiation. By day 80 (D80), organoids developed layered structures enriched with deep‐layer cortical markers CTIP2 and upper‐layer marker SATB2 (Figure 2d), indicating progressive maturation and laminar organization. This is also supported by SOX2 and CTIP2 immunostaining (Supplementary Figure S1c). Due to its large size, it is common for COs in culture to develop a necrotic core (Figure 2d, arrow), consistent with literature on issues with long‐term human CO culture [12, 33, 34].

To model chronic stroke, we employed a photothrombotic cortical infarction paradigm using RB [35, 36], followed by a 10‐day infarct maturation period [26, 27, 28] to establish infarcted tissue with glial scar formation which were visually confirmed under surgical microscope by a pale regions close to the cortical surface (Figure 2e). Immunohistochemistry at day 10 post‐stroke showed robust GFAP+ glial scar formation surrounding the lesion cavity (Figure 2f). This defined injury stage provided a permissive window for surgical intervention. We then established a two‐step transplantation procedure in the chronic stroke model under the direct visualization of a surgical microscope. First, necrotic infarct tissue was removed via stereotaxic aspiration, exposing a well‐defined wound cavity (Figure 2g). Subsequently, COs at the age of D56 post‐differentiation were first split into four pieces (each has a diameter of ∼1 mm) and then gently aspirated into glass pipettes (∼250 *µm* inner diameter opening). Then they were slowly injected into the cleaned infarct space while step‐wise elevation was performed as we previously described [37] (Figure 2h). This surgical approach enabled controlled and reproducible delivery of COs into the lesion site, forming the foundation for assessing post‐transplantation integration and repair.

### 2.3 In Vivo MRI Monitoring of Transplanted COs

**FIGURE 3:**
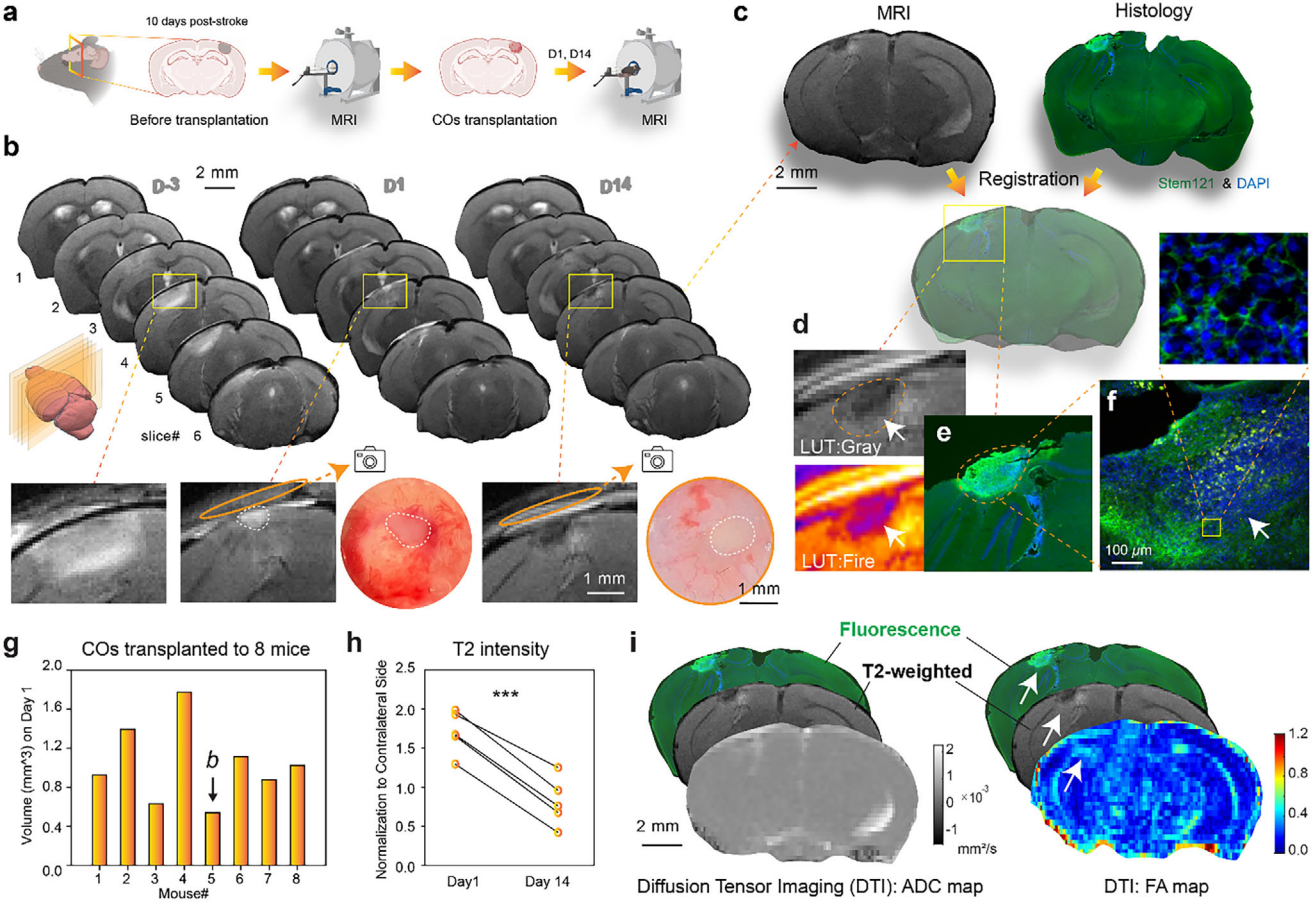
In vivo MRI monitoring of transplanted COs. (a) Schematic illustration of the experimental timeline for MRI scans and CO transplantation. (b) Representative T2‐weighted (T2w) MRI images of a mouse brain at D‐3, D1, and D14. Yellow boxes indicate the magnified regions shown in the top rows. The bottom row displays overhead light microscopy photos of the corresponding brain regions, confirming the presence of the transplanted COs. (c) Co‐registration of MRI and histology images. The T2w MRI image (left) and the corresponding histology image stained for human antigen marker (STEM121, green) and DAPI (blue) (right) are shown for slice #4 at D14. (d) Magnified view of the CO engraftment site from the T2w MRI on D14, showing inhomogeneous T2 hypointensity regions (top, LUT Gray) and corresponding pseudocolored image (bottom, LUT Fire). (e) Corresponding histological image of the CO engraftment site, showing the outline of the implanted CO and viable grafted cells. (f) Higher magnification of (e) with arrows indicating areas of densely packed nuclei and fewer human cells that colocalize with the dark spots in the T2w MRI (arrows in d). The inset shows the morphology of viable grafted cells. (g) Quantification of the volume of implanted COs from 8 mice. *b* indicates the animal shown in b. (h) Quantification of T2w intensity, normalized to the contralateral side, from 5 mice with trackable COs, showing a significant drop in T2w signals from D1 to D14 (*p* = 0.0002, paired t‐test, n = 5 mice). (i) Diffusion Tensor Imaging (DTI) analysis of the CO engraftment site on D14. Top: T2w image with fluorescence overlay. Left: Apparent Diffusion Coefficient (ADC) map. Right: Fractional Anisotropy (FA) map, revealing distinct signal enhancement at the graft‐host interface (white arrowheads).

To assess initial engraftment and monitor the spatial characteristics of transplanted COs in vivo, we employed high‐resolution T2‐weighted (T2w) MRI following transplantation using a 9.4T MRI scanner (Bruker, Ettlingen, Germany). MRI scans were acquired at ‐3, 1, and 14 days post‐transplantation of COs to capture dynamic changes in graft location and property (Figure 3a). On Day 1 post‐transplantation, the graft appeared as a distinct T2‐hyperintense region within the infarct cavity on MRI. Light microscopy images acquired during transplantation showed the organoid in the corresponding cavity location (dashed regions, Figure 3b). On Day 14, the border of COs was not as obvious as on Day 1. To find out the exact border of the graft, we performed immunostaining of CO through human antigen marker (STEM121) and registered the same coronal brain slice (slice#4, Figure 3b) under MRI and histology (Figure 3c), which clearly reveals the outline of implanted CO as well as the viable grafted cells (Figure 3f, inset). Interestingly, the pattern of inhomogeneous T2 hypointensity regions in the CO engraftment site (Figure 3d) matched well with the histology observation (Figure 3e,f): dark spots colocalize with densely packed nuclei with fewer human cells (arrows in Figure 3d,f).

From the 8 mice that received CO transplantation, we drew regions of interests (ROIs) over the graft on T2w images. Relative to the mouse undergoing the same ischemic core removal procedure but without CO transplantation (Supplementary Figure S2a), the implanted COs exhibited high T2w signal on D1 post‐transplantation. Intraoperative surgical microscopy images acquired at the time of transplantation showed the organoid occupying the corresponding cavity location (Supplementary Figure S2b). We then quantified the volume of implanted COs ranged from 0.54 to 1.78 mm^3^, with a mean of 1.0 mm^3^ (Figure 3g). T2 MRI images from representative planes on Day 1 from more animals included in this study are shown in Supplementary Figure S2b,c. We noticed graft‐associated T2w signal from the implanted CO on D14 (Figure 3d, dashed region) was reduced relative to D1 (Figure 3b, D1, slice#4, dashed region). Quantification from 5 mice with trackable COs reveals a significant drop of T2w signals (Figure 3h, *p* = 0.0001, n = 5 mice). This T2 decrease could reflect a reduction in graft water content as acute post‐surgical edema resolves. Because the graft was not always straightforward to delineate on MRI at later time points, we focused on changes in T2‐weighted signal intensity rather than serial MRI‐based volumetric measurement of graft size. For the same reason, interpretation of the Day 14 MRI appearance was based on histological correlation rather than MRI alone.

By Day 14, the graft‐host boundary became less distinct on T2w images, which could indicate some blending of graft with host tissue or simply the resolution of edema. As no clear borders of the CO graft could be revealed under T2w images at D14 post‐transplantation, we performed diffusion tensor imaging (DTI) to generate apparent diffusion coefficient (ADC) and fractional anisotropy (FA) maps (Figure 3i). Co‐registration of fluorescence‐based histology with T2w and DTI images enabled precise localization of the grafts within the infarct cavity. Interestingly, out of the 22 parameters from the DTI analysis result (Supplementary Figure S3a), FA maps revealed a distinct signal enhancement at the graft–host interface (Figure 3i, arrows), a similar pattern was observed in multiple animals (Supplementary Figure S3b). This elevated FA may reflect aligned structures at the graft–host interface, such as axons, glial processes, collagen, or nascent vasculature. Together, these findings demonstrate that DTI offers complementary, noninvasive readouts of graft engraftment and host response—providing structural evidence of early donor‐host interactions and potential neural remodeling induced by CO transplantation. Taken together, these MRI findings provide an important anatomical component of the MIPOT framework, complementing the viability and microscopic information obtained from BLI and TPFM.

### 2.4 Longitudinal Tracking of the Viability of Transplanted COs Through BLI

**FIGURE 4:**
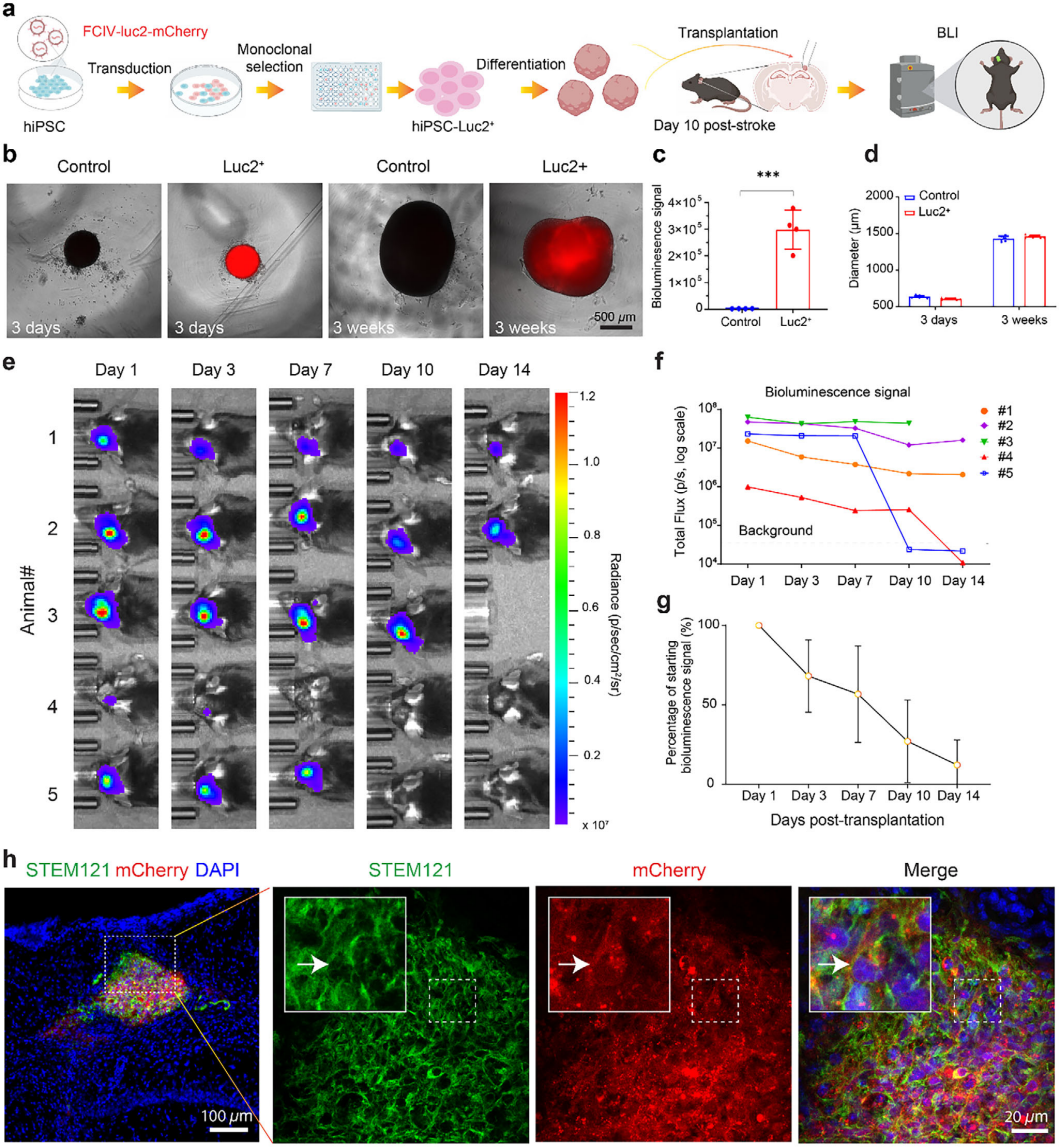
In vivo tracking of hiPSC‐derived cortical organoid transplantation using bioluminescence imaging (BLI). (a) Schematic workflow of generating Luc2‐mCherry dual‐reporter hiPSCs, differentiation into COs, transplantation into stroked mouse brains at 10 days post‐injury, and longitudinal tracking using BLI. (b) Brightfield and fluorescent images showing control and Luc2+ COs at day 3 and week 3 in vitro. Luc2+ organoids exhibit strong mCherry fluorescence without affecting gross morphology. (c) Quantification of luminescence signal confirms significantly higher signal in Luc2+ organoids compared to control organoids (***p = 0.0002, n = 4 COs per group, unpaired t test). (d) Diameter measurements of control and Luc2+ organoids at 3 days and 3 weeks show no significant difference, indicating stable growth kinetics. (e) In vivo BLI images from 5 animals over 14 days post‐transplantation show localized signal from transplanted Luc2+ COs. (f) Longitudinal quantification of total BLI signal per animal. Total flux is plotted on a logarithmic scale. (g) Mean percentage of retained bioluminescent signal relative to day 1 reveals ∼25% of initial signal remains by day 14 (n = 4 animals). (h) Immunofluorescence analysis at endpoint (day 14) confirms survival of grafted cells. Human‐specific STEM121 (green) and mCherry (red) co‐expression within the graft (arrow) validates the in vivo BLI signal and confirms survival of transplanted human cells. Nuclei counterstained with DAPI (blue). All scale bars are as indicated in each panel.

To enable longitudinal, noninvasive assessment of graft viability following CO transplantation, we incorporated BLI as a core component of our MIPOT system. Human iPSCs (hiPSCs) were transduced with a lentiviral construct encoding luciferase (Luc2) and mCherry driven by the constitutive human ubiquitin C promoter [38, 39, 40], followed by monoclonal selection and differentiation into COs for transplantation to mice 10 days after the onset of photothrombosis (Figure 4a). Luminescence reporter system allows longitudinal tracking of CO viability through BLI without the need for invasive procedures. To validate reporter expression, we monitored COs at early (3‐day) and later (3‐week) stages after transplantation. Compared to control organoids, Luc2+ COs showed strong mCherry fluorescence (Figure 4b) and luminescence (Figure 4c) at both time points. Quantification of luminescence confirmed significantly elevated signal in Luc2+ COs versus controls (*p*  = 0.0002, Figure 4c), with comparable diameters across conditions and time points (Figure 4d, *p* = 0.0769, 3 days and 0.0946, 3 weeks). This indicates that the genetic modification associated with lentiviral labeling did not affect growth or differentiation potential of iPSCs as immunostaining COs with Tuji1 at older stages confirm that Luc2+ COs are capable of further neuronal maturation (Supplementary Figure S4a).

Following transplantation into the ischemic cavity after wound cleaning at the chronic phase of stroke (10 days post‐stroke), graft viability was monitored over two weeks using in vivo BLI (Figure 4e). All animals (n = 5 mice) exhibited localized above‐background photon emission from the graft site beginning from day 1 post‐CO transplantation. However, BLI signals declined progressively till 10 days post‐transplantation and stabilized (Figure 4f), with only ∼25% of the initial signal remaining by day 14 (Figure 4g), indicating partial but sustained graft viability during the early post‐transplantation phase. The decline in BLI signal likely reflects reduced graft viability, but we cannot exclude a contribution from temporal changes in luciferin delivery, as D‐luciferin brain distribution is known to be limited by transport‐related factors [41, 42, 43]. We then performed histological analysis at the endpoint to confirm the presence of human cells within the host brain. Immunofluorescence for human‐specific STEM121 (green) revealed grafted CO cells colocalized with mCherry (red) in the graft, validating the accuracy of the BLI signal and confirming survival of a subset of transplanted cells (Figure 4h and Supplementary Figure S4b). To further support the use of BLI as a viability readout, we established an in vitro standard curve showing a strong correlation between luminescence and Luc2‐mCherry+ hiPSC number (R^2^ = 0.991, Supplementary Figure S5a), and performed an endpoint comparison showing a positive relationship between in vivo BLI signal and histologically measured graft area (R^2^ = 0.9753, Supplementary Figure S5b). To further corroborate the main BLI findings, we performed longitudinal BLI in an additional set of transplanted mice (n = 10 mice) and observed a similar overall pattern of progressive signal decline over time (Supplementary Figure S5c,d). Across the full BLI dataset, all transplanted mice (15/15) showed localized above‐background signal on the first timepoint (Day 1 in Figure 4e and Day 0 in Suppl. Figure 5c), indicating successful initial graft delivery. Longitudinal BLI further showed progressive reduction in retained graft‐associated signal over time, with mean signal decreasing to 51% of the start signal at Day 4, 26% at Day 7, 17% at Day 10, and 5% at Day 14 (n = 15 mice, Supplementary Figure S5e). These results demonstrate that BLI within the MIPOT platform offers a sensitive, noninvasive approach to dynamically assess early graft viability after CO transplantation into the chronically ischemic brain.

**FIGURE 5:**
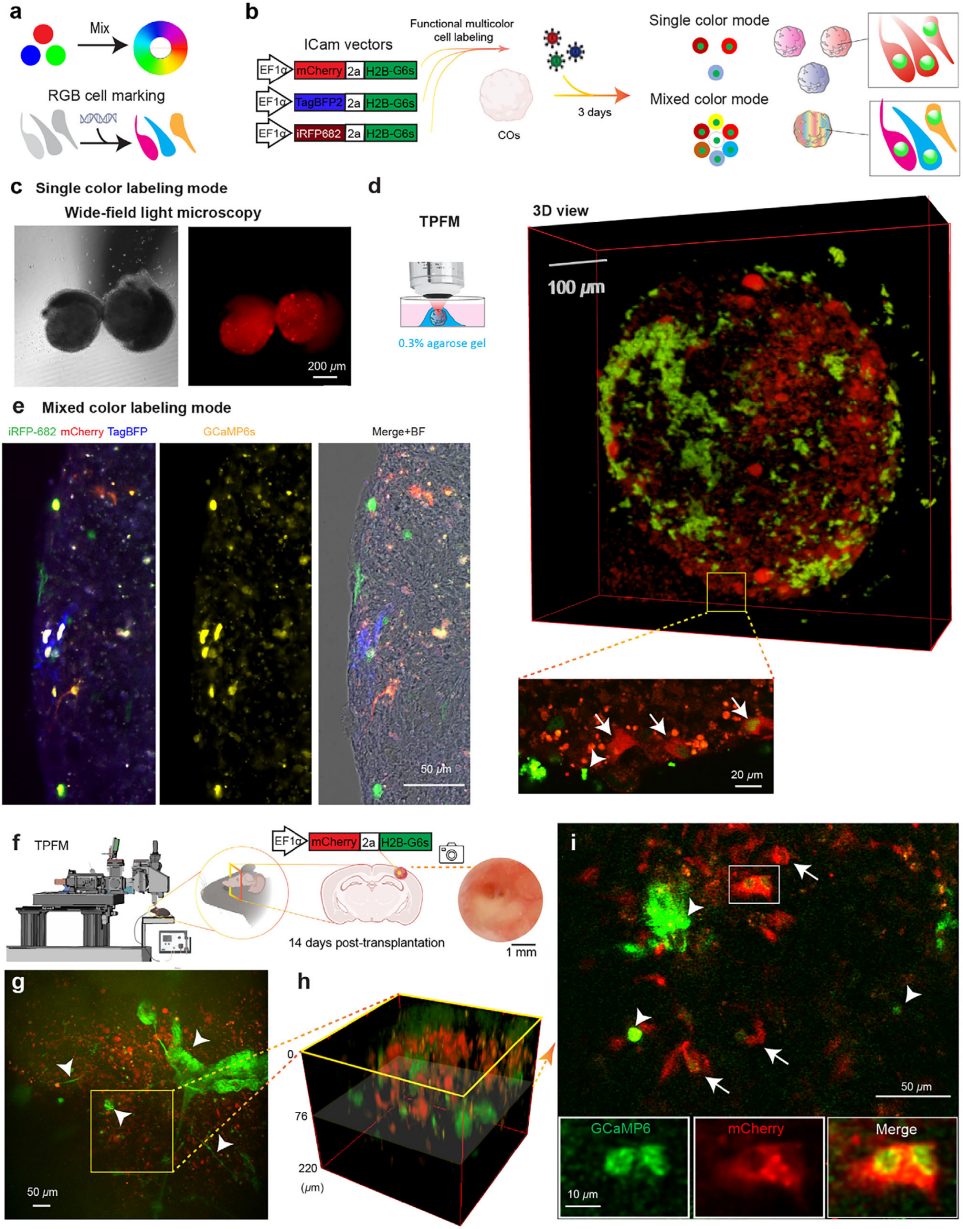
Fluorescent labeling of COs for TPFM. (a) Schematic of RGB cell labeling principle based on the combinatorial expression of three fluorescent proteins (mCherry, TagBFP, iRFP682), enabling a spectrum of colors via mixing. (b) Diagram of ICam vectors used for functional multicolor labeling of human iPSC‐derived (COs). Three separate constructs drive the expression of nuclear H2B‐GFP in tandem with mCherry, TagBFP, or iRFP682. Labeling results in single‐color or mixed‐color modes after 3 days of lentiviral transduction. (c) Wide‐field bright field and fluorescence images of COs labeled in single‐color mode by ICam‐mCherry. (d) TPFM imaging of a CO labeled in single‐color mode by ICam‐mCherry. Left: schematic of in vitro imaging setup. Middle: 3D rendering of the organoid. Right: higher magnification image highlights distinct cells expressing mCherry and G6s (arrows). SHG signals were indicated by arrowheads. (e) Multichannel fluorescence images of a sliced CO labeled in mixed‐color mode, showing mCherry, iRFP682, TagBFP, and GCaMP6s signals. Merge+Brightfield (BF) image confirms multicolor labeling within individual cells. (f) Schematic of in vivo two‐photon imaging of COs grafted into the post‐stroke cortex. At 14 days post‐transplantation, labeled COs are imaged through a cranial window. (g) Representative in vivo two‐photon image showing mCherry‐positive grafted cells (red) and GCaMP6s expression (green). Arrowheads indicate grafted cells. (h) 3D reconstruction of the grafted CO by zooming in a region in (g). (i) The horizontal plane from (h) showing spatial distribution of mCherry and GCaMP6s signals as well as structured SHG signals (arrow heads). Insets show magnified views of the boxed region with individual channels and merged images, highlighting structural features of transplanted cells.

### 2.5 Labeling and High‐Resolution Two‐Photon Imaging iPSC‐COs

To enable high spatial resolution tracking of grafted COs in vivo, we previously established a multicolor fluorescence labeling strategy and integrated it with two‐photon fluorescence microscopy (TPFM), which is widely used for high‐resolution in vivo brain imaging [44, 45, 46, 47]. Our goal here is to monitor both the migration and functional activity of human iPSC‐derived COs after transplantation into the ischemic cortex at the chronic phase of stroke. To achieve this, we utilized the RGB cell marking system, previously developed based on additive color mixing principles to distinguish individual cells by unique fluorescent hues (Figure 5a), and recently optimized it with our newly developed ICam vector system for functional labeling of neurons [37, 48]. We constructed three ICam vectors, each co‐expressing a distinct fluorophore—mCherry, TagBFP, or iRFP‐682—linked via a self‐cleaving 2A peptide to H2B‐GCaMP6s (H2B‐G6s), a nuclear‐localized calcium indicator (Figure 5b). COs were labeled using either single color mode, where one cytosolic fluorophore was introduced (Figure 5b,c), or mixed color mode, where a combination of vectors was used to generate a spectrum of color‐coded cells in 7 patterns (Figure 5b, right). In the single‐color mode, wide‐field fluorescence imaging showed distinct red and green fluorescent nuclei within the COs labeled with ICam‐mCherry, confirming efficient gene delivery and expression (Figure 5c). However, under wide‐field fluorescence microscope, due to the scattering nature of the CO, it is challenging to obtain high‐resolution images (Supplementary Figure S6a). In contrast, TPFM enables subcellular resolution imaging of the live CO (Figure 5d), revealing the nucleus‐located G6s and cytosolic localization of mCherry (arrows in Figure 5d) as in the single‐color condition, the lentiviral construct co‐expresses cytoplasmic mCherry and nuclear‐localized H2B‐GCaMP6s, allowing the same grafted cell to be visualized with red cytosol and green nuclei under 1000 nm two‐photon laser excitation. Strong second‐generation harmonic (SHG) signal was detected in the live CO (Figure 5d, arrow heads in the inset). In the mixed color mode, individual cells within the same organoid could be uniquely identified by their color hues from each of the three cytosolic FP and the nuclear G6s as revealed by the presence of colorful cells in the CO transduced with a 1:1:1 ratio mixture of the ICam vectors in terms of infectious unit (Figure 5e). Importantly, these FPs could also be unmixed by special acquisition settings under TPFM [37], enabling multicolor labeling of CO for potential functional cell tracking (Supplementary Figure S6b).

To explore the feasibility of in vivo imaging CO integration after transplantation into the chronically infarcted cortex, we performed intravital TPFM imaging 14 days post‐transplantation (Figure 5f). COs were pre‐labeled with ICam‐mCherry at 7 weeks post‐differentiation and grafted at 8 weeks post‐differentiation to chronic stroke in a manner similar to previous sessions. Under TPFM, CO cells were clearly identifiable within the stroke cavity in vivo (Figure 5g–i). Interestingly, different from the in vitro CO, the SHG signal exhibited thick fiber‐like 3‐dimensional structures (Figure 5g–i, arrows). High‐resolution imaging revealed co‐expression of mCherry and GCaMP6s, confirming the persistence of graft cells carrying the structural and calcium‐reporter constructs at the transplantation site (Figure 5i, arrows and insets). We also performed longitudinal in vivo cell tracking over 4 h at 30 min intervals, enabling re‐identification of individual grafted CO cells and reconstruction of their trajectories over time (Supplementary Figure S7a–d). Within this imaging window, the tracked cells exhibited only modest displacement. In one representative cell, longitudinal imaging showed fluctuations in H2B‐GCaMP6s intensity over time relative to the mCherry signal (Supplementary Figure S7d), indicating that reporter levels could vary during repeated imaging. However, short‐term time‐lapse imaging did not reveal robust spontaneous calcium transients across the recorded cells (Supplementary Figure S7e–g). These datasets were acquired at early post‐transplantation time points (day 3 in mouse 2 and day 6 in mouse 5), which may partly account for the limited cell movement and the lack of consistent calcium activity. Together, this system—integrating RGB‐based multicolor labeling, genetically encoded calcium indicators, and intravital two‐photon imaging—provides a robust platform to dynamically monitor cell fate and activity following CO transplantation in stroke.

### 2.6 A Removable 3D‐Printed Head‐Fixation System Enables Same‐Animal MRI, BLI, and TPFM

**FIGURE 6:**
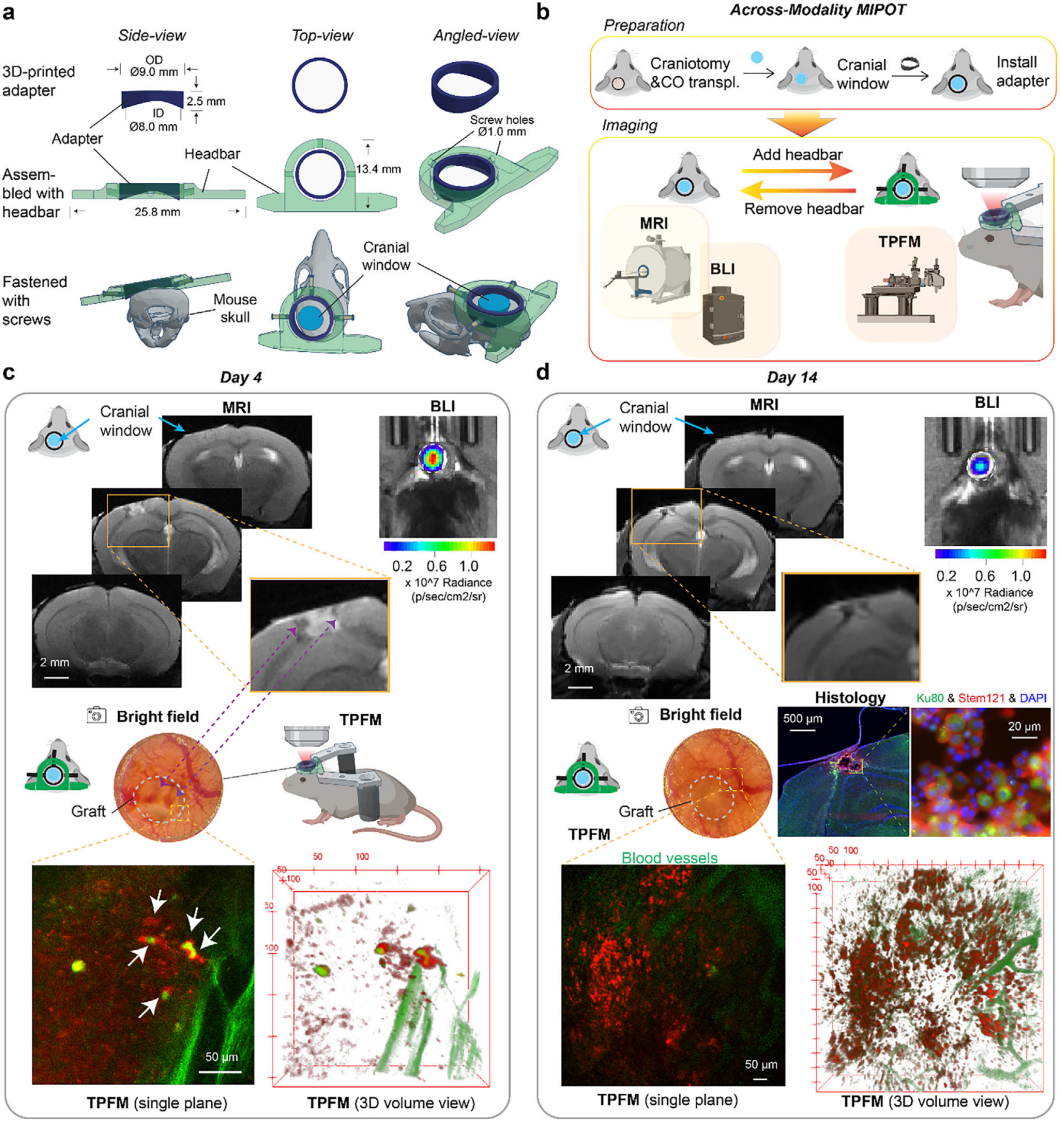
Cross‐modality imaging of transplanted COs using a removable 3D‐printed head‐fixation system. (a) Structural design of the two‐component fixation system, consisting of a skull‐mounted adapter and a detachable headbar. Individual components, assembled views, and positioning over the cranial window are shown. (b) Schematic of the sequential imaging workflow. Following CO transplantation and cranial window implantation, animals undergo MRI and BLI with the headbar removed, followed by attachment of the removable headbar for TPFM. (c,d) Representative same‐animal imaging from one of two mice subjected to sequential MRI, BLI, and TPFM after transplantation, shown at day 4 (c) and day 14 (d). For each time point, the figure includes coronal MRI images and corresponding enlarged views of the transplant site, BLI, bright‐field imaging through the cranial window, and TPFM. A 3D‐rendered TPFM image is shown in c, and terminal histology is included in (d) with brain slices immunostained with human nuclei marker ku80 (green), human cell cytosol marker stem121 (red), and DAPI. Dashed outlines mark the graft region. Purple arrows mark focal surface spots seen in the bright‐field images. Signal identities and scale bars are indicated in the corresponding panels.

To enable serial multimodal imaging in the same animal, we developed a two‐component plastic head‐fixation system consisting of a skull‐mounted 3D‐printed adapter and a removable headbar (Figure 6a). This design avoids the MRI incompatibility of conventional metal headbars while still allowing rigid head fixation for two‐photon imaging. After organoid transplantation and cranial window implantation, mice can undergo MRI and BLI with the headbar removed, and the removable headbar can then be attached to the adapter for subsequent TPFM imaging (Figure 6b).

To test whether this preparation supports sequential cross‐modality imaging in the same animal, we performed MRI, BLI, and TPFM in mice transplanted with COs expressing luciferase and labeled with ICam‐mCherry, thereby enabling tracking by both BLI and TPFM. A representative example imaged on day 4 and day 14 after transplantation is shown in Figure 6c,d. With the headbar removed, the animal first underwent MRI and BLI. T2‐weighted MRI revealed a high signal from the transplantation site at early phase of transplantation (Figure 6c, MRI), which drops significantly at Day 14 (Figure 6d, MRI), consistent with our results from Figure 3h. From coronal planes of MRI images, the cranial window is also clearly visible in either timepoint (Figure 6c,d, cranial window). BLI shows substantial luminescence signal at day 4 post‐transplantation (total flux: 4.39 × 10^7 photons/s) indicating large number of viable cells from the graft (Figure 3c, BLI), which underwent a substantial drop at Day 14 (total flux: 0.90 × 10^7 photons/s, decreased by 79.4%), consistent with our BLI results (Figure 4 and Supplementary Figure S5). After MRI and BLI, the removable headbar was installed and fastened to enable stable imaging of the mouse brain under light microscopy and subsequent TPFM. Cranial window enables clear visualization of the grafted CO (outlined by gray dots) under direct visualization through stereoscope, revealing two potential bleeding spots that correspond to the MRI T2* images (Figure 6c, purple arrows). TPFM at day 4 resolved graft‐derived cells at subcellular resolution, showing mCherry‐labeled cytosol and nuclear GCaMP6s signal. By day 14, TPFM detected prominent superficial blood vessels overlying the graft site, whereas only sparse graft‐derived fluorescent cells were visible. Imaging depth at this stage was limited to approximately 200 µm because of reduced cranial window quality. Terminal histology with Ku80 & stem121 staining confirmed the presence of surviving human graft cells and aided interpretation of the corresponding MRI features (Figure 6d).

Together, these findings demonstrate that the two‐component head‐fixation system enables sequential MRI, BLI, and TPFM in the same animal, providing complementary information on graft location, viability, and microscopic appearance over time. This multimodal approach provides a foundation for future spatiotemporal studies of graft survival, morphology, and host interaction in the living brain.

### 2.7 Histology Evaluation of Implanted COs in Chronic Stroke

**FIGURE 7:**
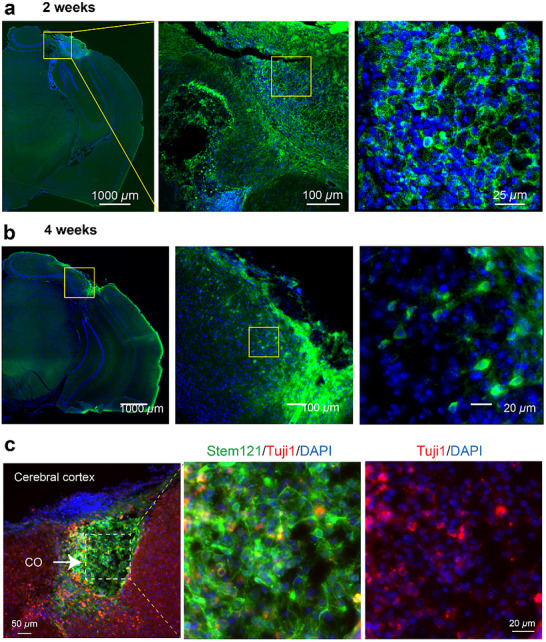
Histological assessment of CO graft persistence in chronic stroke cavities. (a, b) Representative immunofluorescence images of graft‐derived human cells in the intended cortical graft region at 2 weeks (a) and 4 weeks (b) post‐transplantation. Sections were stained for the human‐specific marker STEM121 (green) and DAPI (blue). White arrows indicate STEM121‐positive graft‐derived cells. Scale bars: 1000 µm, 100 µm, and 25 µm in (a); 1000 µm, 100 µm, and 20 µm in (b). (c). Representative images of TUJ1/STEM121 immunostaining in the intended cortical graft region at 4 weeks post‐transplantation. TUJ1 (red) marks neuronal marker expression, STEM121 (green) identifies graft‐derived human cells, and DAPI (blue) labels nuclei. Limited TUJ1/STEM121 overlap was observed within the cortical graft region.

Using the MIPOT platform, we tracked graft dynamics through BLI during the early post‐transplant phase, revealing an initial decline and gradual stabilization trend in graft‐associated BLI signals during the first two weeks (Figure 4e–g). This pattern is consistent with the vulnerable window reported for transplanted cells and brain organoids, in which graft survival often stabilizes after approximately 2 weeks post‐engraftment [49, 50, 51]. To extend these observations, we analyzed brains collected at 2‐ and 4‐weeks post‐transplantation. Immunostaining for the human‐specific antigen STEM121 confirmed the presence of COs at both time points, indicating limited persistence of transplanted cells (Figure 7a,b). Additional co‐staining for the human nuclear marker Ku80 and STEM121 provided supporting evidence for the human identity of remaining graft‐derived cells at 4 weeks post‐transplantation (Supplementary Figure S8a,b). At 4 weeks post‐transplantation, TUJ1/STEM121 immunostaining in the intended cortical graft region showed sparse TUJ1 colocalization with STEM121‐positive cells, consistent with limited neuronal marker expression within the current observation window (Figure 7c). In a small subset of animals (n = 4/14), graft‐derived cells were incidentally detected outside the intended cortical lesion cavity, including in hippocampal regions (Supplementary Figure S9a–g). Some hippocampal graft‐associated cells displayed extended neurite‐like morphology and TUJ1 expression. Because these cases resulted from unintended graft placement and were not part of a planned or controlled hippocampal transplantation experiment, these data are presented only as limited descriptive observations. They should not be interpreted as evidence that hippocampal regions support greater graft survival, neuronal differentiation, or maturation than the post‐stroke cortical cavity.

## 3 Discussion

In this study, we established a Multimodal Imaging Platform for Organoid Tracking (MIPOT) that enables longitudinal, multi‐scale assessment of hiPSC‐derived COs transplanted into chronic stroke lesions. By combining intraoperative microscopy, high‐field MRI, BLI, and TPFM, MIPOT allows us to confirm initial graft placement, longitudinal monitoring of viability‐associated signal dynamics, and high‐resolution visualization of graft‐derived cells in vivo. We observed that CO grafts filled the lesion cavity immediately after delivery but experienced a rapid viability decline over the first ∼10 days, with only around a quarter of the initial cell survival by week 2. This early decline is consistent with prior reports that vascularized organoids shrink during the first 10–14 days before host perfusion is established [20, 49, 52]. At 4 weeks post‐transplantation, a small number of human graft‐derived cells were still detectable in the cortex, indicating limited persistence of transplanted cells within the chronic injury environment, which is more hostile than developing [49, 53] or non‐prior injury rodent brains [20, 52] in previous organoids transplantation studies. Together, our work establishes a multimodal framework for peri‐transplantation tracking of organoid grafts in chronic stroke lesions and identifies key technical and biological challenges, including limited graft persistence and the need for improved strategies to support long‐term survival and maturation.

Chronic phase stroke is featured by the formation of the ischemic core surrounded by glial scar, which prevents the integration of implanted cells or tissues. Previous work has shown that photothrombotic lesions stabilize between 7–10 days, with glial scar formation, hemorrhage resolution, and lesion volume plateauing during this subacute‐to‐chronic transition [26, 27, 35]. We reason that by removing the well‐formed ischemic core, we could provide the grafted COs a clean parenchyma surface for them to integrate, which has been demonstrated in many prior studies in non‐stroke animals [52, 53, 54], although this benefit must be balanced against procedural risks such as bleeding and additional tissue injury. Using our previously designed transplantation approach, pulse‐elevation [37], we achieved consistent MRI‐confirmed initial placement of CO fragments within the lesion cavity in all animals examined (8 out of 8, Figure 3). However, this initial placement should be distinguished from sustained graft survival, which remained limited and requires further optimization. This is a solid step forward as consistency and reliability has been a major challenge for the transplantation of brain organoids due to the finicky nature and the lack of well‐documented methods in many previously published papers. The efficacy of this ischemic‐core‐removal and ensuing CO transplantation approach is demonstrated by the imaging and histology results showing viable cells at 2 or 4 weeks post‐transplantation (Figure 7a,b). In a small subset of animals (n = 4), graft‐derived cells were incidentally observed outside the intended cortical lesion cavity, including in hippocampal regions. Because these cases were not generated through a planned or controlled hippocampal transplantation experiment, they are presented as descriptive observations only. Although some hippocampal graft‐associated cells displayed more extended neurite‐like processes and neuronal marker expression, the observed differences between post‐stroke cortex and hippocampus should be considered preliminary and descriptive and should not be interpreted as evidence that the hippocampus provides a more favorable environment for CO maturation. A dedicated study with controlled targeting, larger cohorts, and functional analyses would be required to evaluate site‐dependent graft outcomes. To our knowledge, we are the first to advocate the removal of ischemic core in chronic stroke and using the resulting cavity for the accommodation of organoids for stroke treatment. Previous work on transplanting CO for stroke treatment focused on the acute or subacute phase of stroke [24] or directly inject the CO into the junction of the infarct core and the peri‐infarct zone of stroke [23] (See Supplementary Table S1 for comparison of our work with previous relevant studies).

Most previous approaches for observation of transplanted brain organoids rely on single modalities or terminal histology as readout of brain organoids transplantation to lesioned [20, 21, 22, 23, 24] or developing animal brains [49, 53]. In one elegant study, multimodal monitoring of transplanted COs in the mouse brain was performed by combining two modalities: TPFM and microelectrode arrays for longitudinal tracking of COs^55^. The novelty of MIPOT is not longitudinal graft tracking itself. Rather, the unique contribution of MIPOT lies in the integration of surgical microscopy, MRI, BLI, and intravital TPFM into a complementary workflow for monitoring human CO transplantation in a chronic stroke cavity model. This combination provides cross‐scale and cross‐modality readouts of initial graft placement, graft‐associated structural changes, viability‐associated signal dynamics, and cellular‐resolution visualization within the same experimental framework.

High‐field MRI provided a noninvasive window into graft anatomy. Although previous studies have used MRI to monitor transplanted organoids at later stages, such as from two months after transplantation onward [53], here we incorporated MRI into a multimodal platform together with BLI and TPFM in a chronic stroke model. We also initiated MRI during the peri‐transplantation period (defined here as Day −7 to Day +7 relative to grafting) to capture early structural changes after implantation. To our knowledge, this is the first use of MRI to evaluate organoid transplantation in the context of stroke therapy. In our hands, COs appeared as distinct T2w hyperintensities in the lesion cavity on Day 1 post‐transplantation (Figure  3b), supporting MRI‐based confirmation of initial graft placement. Over 2 weeks, we observed a significant decrease in T2w signal (Figure  3h), which likely reflects reduced water content and compaction as edema resolves. We also collected diffusion MRI (DTI) metrics at 2 weeks and found elevated fractional anisotropy at the graft–host interface (Figure  3i). These MRI readouts (anatomy, volume, diffusion) provide quantitative gauges of engraftment, building on in vivo MRI studies mapping stem cell migration post‐stroke [55]. Importantly, histology registered to MRI confirmed that T2w hypointensities corresponded to densely packed nuclei (Figure  3d–f), which may be the result of inflammatory cell invasion of the graft [56]. In sum, MRI supported assessment of initial graft placement and provided early information on graft‐associated structural changes, demonstrating the value of combining anatomical imaging with histological correlation.

To noninvasively measure graft viability, we labeled COs with a luciferase reporter and performed serial BLI (Figure  4e and Supplementary Figure S5c). This revealed that most photon emission originated from the transplant site, and that luminescence declined markedly within the first 10 days (Figure  4f–g and Supplementary Figure S5d,e). By day 14 the signal had plateaued at around a quarter of its peak. This finding is consistent with a general “vulnerable period” for grafts: other studies have found that stem‐cell survival often stabilizes after ∼2 weeks post‐transplant [20, 49, 52]. Crucially, our histology confirmed that the remaining bioluminescent signal was indeed from viable human cells (STEM121+ and mCherry+; Figure  4h). Thus, BLI in MIPOT provided a quantitative in vivo measure of graft survival over time. As a method, BLI is uniquely sensitive for tracking transplanted cells: it has been widely applied to follow cell therapy in live animals [38, 39, 57]. Our data demonstrate that only a fraction of transplanted COs persist beyond the early post‐transplantation period in chronic stroke, emphasizing the need for strategies (e.g. neurotrophic or angiogenic support) to improve survival. In the context of our work, the BLI signal decline defines the early graft‐stabilization window, which complements the anatomic changes seen by MRI and may help guide the timing of future interventions. Although graft survival remained limited with the current transplantation methodology, the primary objective of this study was to establish MIPOT as a multimodal platform for longitudinal in vivo monitoring of graft fate rather than to define an optimized transplantation protocol. From this perspective, the observed early decline in BLI signal is itself informative, as it delineates a vulnerable post‐transplantation window and reveals the extent of inter‐animal variability. These capabilities position MIPOT as a useful framework for future studies aimed at improving graft survival, migration, and host interaction.

We further exploited MIPOT's multiscale capability by implanting fluorescently labeled COs and performing intravital TPFM. Using an RGB viral labeling system co‐expressed with a nuclear calcium indicator (called ICam [37]), we were able to tag individual CO cells with color and image them at high resolution through TPFM (Figure  5d,e). TPFM at 14 days post‐transplant revealed labeled graft CO cells in situ (Figure  5g–i). Interestingly, we observed thick SHG signals in the fiber structure within the graft, possibly originating from collagen. This could be an indication of extracellular matrix remodeling or vascularization, which has been previously reported in transplanted brain organoids to the mouse brain [49, 52, 54]. Importantly, we directly visualized graft cell morphology at subcellular resolution: nuclei exhibited the calcium indicator signal and cytosol the chosen fluorophore, confirming that transplanted neurons remained intact and potentially active (Figure  5i). Thus, TPFM completes the imaging cascade: it allows us to link graft viability (from MRI/BLI) with cellular phenotypes and potential interactions in the living brain. These data also imply host remodeling around the graft; the prominent SHG signal suggests host‐derived collagen or myelin deposition, a phenomenon worth investigating for its impact on graft integration. Our proof‐of‐feasibility cell tracking data indicates that individual graft‐derived cells can be re‐identified and tracked over hours in vivo. However, these tracking data should not be interpreted as evidence of functional activity or host‐graft circuit integration. Extension of this approach to days or weeks will require improved long‐term window quality, reliable field re‐registration, and more robust functional readouts.

Despite demonstrating the feasibility of multimodal imaging to track CO transplantation in chronic stroke, our study has several limitations. First, our transplantation method involved splitting organoids and delivering them through thick glass pipettes similar to needle [53]. While this approach allows better cavity filling and potentially enhances graft‐host interface integration—crucial for stabilizing the cavity and preventing bleeding—it may introduce greater physical stress to the cells, potentially reducing viability compared to transplanting intact organoids [20, 54]. Future studies could directly compare these approaches to evaluate the trade‐offs between anatomical fit and graft survival. Second, although we used cyclosporin A for immunosuppression based on precedent in recent literature [20], long‐term studies would benefit from using genetically immunodeficient animals [52, 53, 54] to ensure consistent suppression of host immune responses over extended periods. Third, although this 2 to 4‐week analysis window reveals crucial dynamics, longer‐term studies (e.g., ≥12 weeks) are needed to evaluate synaptic connectivity, electrophysiological maturity, and behavioral impact. This is particularly important for human brain organoids as it takes many months for human neurons to reach full maturity, as shown in prior studies [58]. Fourth, future work should incorporate vascular imaging agents and functional reporters to assess graft perfusion and neuronal activity over time. Advanced CEST MRI could be explored for label‐free tracking of the graft [59]. Fifth, behavioral assessment of graft efficacy was not performed in this initial study, given the short timeline and immunosuppressive regimen; our aim here was to establish the MIPOT imaging platform and observe integration phenotypes. Future studies with longer‐term graft survival will include behavioral tests to evaluate functional recovery and to link cellular outcomes with functional recovery. Sixth, although TPFM enabled visualization and short‐term re‐identification of graft‐derived cells, robust spontaneous calcium activity was not consistently detected within the current short survival window. Therefore, the present study does not establish functional maturation, neuronal network activity, or host‐graft circuit integration. A further limitation is that the COs were divided before transplantation, a practical approach that has also been used in prior organoid transplantation studies [60, 61, 62], but one that may partially disrupt pre‐formed structures such as rosettes or primitive laminar organization. In addition, because only four animals showed graft involvement in the hippocampus, observed differences in neuronal maturation between post‐stroke cortex and hippocampus should be considered preliminary and descriptive rather than a basis for quantitative conclusions regarding graft efficiency or survival in that location.

An additional consideration in applying MIPOT is how to balance reporter burden against multimodal completeness. MIPOT can be implemented in two complementary modes. In one mode, individual imaging modalities are applied in separate cohorts, thereby minimizing the degree of reporter engineering in each graft and reducing the possibility that exogenous labels influence cell phenotype, viability, or maturation. This modality‐specific strategy is advantageous when the priority is to obtain the least perturbed readout from a given imaging approach. In the second mode, modalities can be combined in the same animal, as demonstrated here by sequential MRI, BLI, and TPFM. This cross‐modality implementation enables direct comparison of anatomical, viability, and microscopic features within the same graft over time, providing a more integrated assessment of transplantation outcome. The trade‐off is that same‐animal multimodal experiments may require more extensive reporter expression and therefore carry a greater risk of biological perturbation. MIPOT thus provides a flexible framework in which investigators can choose either a lower‐burden modality‐specific design or a more comprehensive cross‐modality design according to the goals of the study. These capabilities establish MIPOT as a flexible platform for future mechanistic studies of organoid transplantation and for the preclinical optimization of organoid‐based therapies for chronic stroke. Although demonstrated here in a chronic stroke model, the MIPOT framework should be broadly adaptable to other in vivo settings involving organoid transplantation.

## 4 Materials and Methods

### 4.1 Human Induced Pluripotent Stem Cells Culture

Human induced pluripotent stem cells (hiPSCs, line name KOLF2.1J, product code JIPSC001000, JAX IPSC catalog, RRID:CVCL_B5P3, XY karyotype, male) were cultured as previously described [63], with minor modifications. Cells were maintained under feeder‐free conditions in mTeSR Plus medium (STEMCELL Technologies, Canada, Cat#100‐0276) on vitronectin‐X(STEMCELL Technologies, Canada, Cat#7180)‐coated plates. hiPSCs were passaged every 4–6 days at a 1:10 ratio using 0.5 mM EDTA (ThermoFisher, 440 Cat#25200056). For reseeding, cells were plated in vitronectin‐XF–coated 6‐well plates, and 10 µM Y‐27632 (ROCK inhibitor; STEMCELL Technologies, Canada, Cat#72304) was added to the mTeSR medium for the first 24 h post‐passage to enhance cell survival. All hiPSCs used in this study exhibited typical pluripotent morphology and marker expression, with no signs of spontaneous differentiation. All hiPSC‐related procedures were approved by the university's ethics committee and complied with established ethical standards for human stem cell research.

### 4.2 Lentiviral Transduction and Clonal Selection of hiPSCs

Human iPSCs were transduced with the lentiviral vector pLV‐FCIV‐luc2‐cherry. Briefly, cells were dissociated using 0.5 mM EDTA and resuspended at a density of approximately 1 × 10^5^ cells in 0.5 mL mTeSR Plus medium supplemented with polybrene (5 µg/mL), ROCK inhibitor (Y‐27632, 10 µM), and 10 µL of lentiviral particles. Cells were incubated overnight at 37°C, after which the medium was replaced with fresh mTeSR Plus.Two days post‐infection, cells were seeded by limiting dilution into three 96‐well plates at a density of ∼150 cells per plate. Clones expressing the reporter (cherry fluorescence) were identified under a fluorescence microscope, and wells containing single fluorescent‐positive colonies were selected. After 7 days of culture in 96‐well plates, selected clones were expanded into 24‐well plates for further propagation and validation.

### 4.3 Generation of Human COs

Generation of human COs was performed based on the previously reported protocols [31, 32] Briefly, feeder‐free hiPSCs were dissociated using Accutase (Sigma, Cat#A6964) and seeded at 9,000 cells per well into low‐attachment 96‐well U‐bottom plates in mTeSR plus supplemented with 20 µM Y‐27632. From day 1 to 5, spheroids were cultured in Essential 6 Medium (Gibco, Cat#A1516401) containing 5 µM dorsomorphin (Sigma, Cat#P5499) and 10 µM SB‐431542 (Tocris, Cat#1614). On day 6, spheroids were transferred to neural medium (NM) consisting of Neurobasal‐A (Gibco, Cat#10888) supplemented with B27 minus vitamin A (Invitrogen, Cat#12587010), GlutaMAX (Invitrogen, Cat#35050‐061), and penicillin‐streptomycin (Sigma, Cat#P4333), along with 20 ng/ml bFGF (Peprotech, Cat#100‐18B) and 20 ng/ml EGF (Peprotech, Cat#100‐15), and cultured on an orbital shaker at 60 rpm. On day 25, organoids were transferred to low‐attachment 24‐well plates and maintained in neural medium supplemented with 20 ng/ml BDNF (Peprotech, Cat#450‐02) and 20 ng/ml NT‐3 (Peprotech, Cat#450‐03). Medium was refreshed daily or every other day as appropriate.

### 4.4 Lentiviral Transduction of Human COs

Lentiviral transduction of hCOs was performed as previously described [64], with minor modifications. Briefly, day 40–45 hCOs were individually transferred into 1.5‐ml Eppendorf tubes containing 100 µl of NM supplemented with 8–10 µl of high‐titer lentiviral vector (LV‐hygro‐ef1a‐iRFP682‐nes‐h2bG6s, LV‐hygro‐ef1a‐Cherry‐nes‐h2bG6s, LV‐hygro‐ef1a‐TagBFP2‐nes‐h2bG6s, ∼10^9^ TU ml^−^^1^; Vector Builder). Organoids were incubated at 37 °C for 45 min in a humidified incubator. Following transduction, each organoid was transferred into a well of a low‐attachment 96‐well plate containing an additional 100 µl of fresh NM and incubated overnight at 37°C, 5% CO_2_. The next day, organoids were washed once with 1 ml of fresh NM and transferred into low‐attachment 24‐well plates containing 1 ml of fresh NM per well. Organoids were maintained in culture with medium changes every 2–3 days. Transgene expression was monitored by fluorescence microscopy beginning 3–5 days post‐infection. Robust expression was typically observed between days 5 and 7 and continued to increase over the following week. Only organoids displaying widespread expression were selected for downstream applications, including transplantation within 14 days post‐infection.

### 4.5 Mice and Grouping

All experimental protocols at the University of Maryland, Baltimore, were conducted according to the National Institutes of Health guidelines for animal research and approved by the Institutional Animal Care and Use Committee at the University of Maryland, Baltimore. Mice were group housed with littermates until craniotomy surgery, after which they were singly housed. Mice were maintained on a 12–12‐h (6 a.m.–6 p.m.) light–dark cycle. 3–4 months old male C57BL/6J mice (25‐30 g, Institutional VR breeding) were used in this study. All mice were anesthetized with isoflurane during surgery and live imaging assays in this study (Vetone, 4–5% for induction and 1.5% for maintenance). Grouping of animals is listed Supplementary Table S2. MRI was performed on 25 mice. BLI was performed on 17 mice. TPFM was performed on 5 mice.

### 4.6 Photothrombotic Stroke Model

Focal cortical infarcts in the motor cortex were induced using the photothrombotic stroke model, as described by Labat‐Gest and Tomasi [36]. Briefly, mice were anesthetized and secured in a stereotaxic frame (RWD, Sugar Land, TX). A midline scalp incision was made, and the connective tissue was carefully removed to expose the intact skull surface. The photosensitive dye Rose Bengal (100 mg/kg body weight) was administered via intraperitoneal injection. A fiber optic cable connected to a cold light source was positioned 2.5 mm lateral to the midline and 1.4 mm anterior to bregma, targeting the motor cortex based on stereotaxic coordinates. 5 min after dye injection, the targeted area was illuminated with a 532 nm laser for 15 min to induce a localized stroke (∼1 to 1.5 mm diameter) in the left cortex. Following the procedure, mice were returned to their cages and provided with moist food to support post‐operative feeding. The photothrombotic infarct size was verified by MRI 3 days after stroke, typically showing a lesion cavity of ∼1–3 mm^3^ in volume.

### 4.7 Organoid Transplantation

CO transplantation was performed based on previously described protocols [52, 53] with slight modifications. Briefly, 10 days after stroke induction, a circular craniotomy (3 mm in diameter) was performed by drilling into the skull over the infarcted area, and the lesion was aspirated using vacuum suction to expose the cavity. One CO (∼2 mm in diameter) was transferred onto a sterile parafilm, and excess medium was carefully removed. The organoid was then quartered along the midline using a sterile surgical blade. A 250‐µm diameter glass pipette with a 45° beveled tip, connected to a 10‐µL Hamilton syringe, was used for transplantation. One quarter of the organoid was gently loaded into the distal tip of the pipette. The syringe was mounted onto a microsyringe pump coupled with a stereotaxic manipulator. To calibrate the injection depth, the pipette tip was gently aligned with the surface of the cavity opening so that it was flush with the cavity edge; this position was defined as z = 0 and used as the reference for all subsequent depth measurements. The organoid delivery method followed a previously established protocol with minor modifications. The injection was performed at three discrete depths: 1 µL was delivered at a depth of 1 mm, followed by a 1‐min pause. The pipette was then retracted to 700 µm, where a second 1 µL was injected, again followed by a 1‐min pause. Finally, the remaining organoid volume was injected at 500 µm depth, followed by a 3‐min pause to allow for tissue accommodation. The pipette was then slowly withdrawn at a controlled speed of 0.2–0.5 mm/min to minimize tissue disruption. Throughout the surgical procedure, the lesion site was continuously irrigated with sterile saline to maintain a clear, blood‐free cavity. Hemostasis was achieved using a sterile gelatin sponge, which also absorbed excess blood and fluid. Following organoid implantation, the graft was covered with either a 3.5‐mm glass coverslip or a layer of sterile plastic wrap to establish a cranial window. The implant was sealed using surgical adhesive. The scalp was then either sutured closed or, alternatively, a custom‐designed headbar (for single‐modality TPFM) or a 3D‐printed plastic headbar and adapter (for cross‐modality MIPOT) was attached to the skull using dental cement to allow for future head fixation during imaging. Upon completion of surgery, animals received analgesia via intraperitoneal injection of carprofen (5 mg/kg, RimadyI). Mice were placed in a temperature‐controlled recovery chamber and subsequently returned to their home cages. Immunosuppression was administered daily following transplantation with cyclosporin A at a dose of 10 mg/kg [65, 66].

### 4.8 3D‐Printed Adapter/Headbar Components

The removable 3D‐printed head‐fixation system was fabricated from STL design files using a Bambu Lab X1‐Carbon 3D printer with polylactic acid (PLA) filament, a 0.4 mm nozzle, and a 0.20 mm layer height. The adapter and headbar components measured approximately 9.0 × 9.0 × 2.5 mm and 25.8 × 13.4 × 2.5 mm, respectively. The STL design files are available at https://github.com/liangy10/MIPOT.

### 4.9 Tissue Collection and Immunofluorescence Staining

Animals were deeply anaesthetized and transcranial perfused with phosphate‐buffered saline (PBS; Gibco, Cat#18912), followed by 4% paraformaldehyde (PFA in PBS; Sigma–Aldrich, Cat#P6148). Brains containing transplanted organoids were post‐fixed overnight at 4°C in 4% PFA and cryoprotected in 30% sucrose (Sigma‐Aldrich, Cat#S9378) in PBS for 48–72 h. Organoids were fixed separately in 4% PFA for 2 h at room temperature and cryoprotected in 30% sucrose for 24 h. Tissues were embedded in OCT compound (brains: Tissue‐Tek OCT Compound 4585, Sakura Finetek; organoids: 1:1 mixture of 30% sucrose and OCT), and coronally sectioned using a cryostat (CryoStar NX50) at 30 µm thickness (brains) or 20 µm (organoids). Cryosections were washed in PBS and incubated for 1 h at room temperature in blocking solution containing 10% normal bovine serum albumin (BSA; Sigma‐Aldrich, Cat#A9647) and 0.3% Triton X‐100 (Sigma‐Aldrich, Cat#SLCD3084) in PBS. Sections were then incubated overnight at 4°C with primary antibodies diluted in blocking solution. The following primary antibodies were used: anti‐Stem121 (mouse, 1:500; Y40410, Takara Bio), anti‐SOX2 (rabbit, 1:300; AB 5603, Millipose), anti‐Nanog (rabbit, 1:200; ab 80892, Abcam), anti‐CTIP2 (rat, 1:200; MABE1045, Sigma‐Aldrich), anti‐SATB2 (mouse, 1:50; ab51502, Abcam), anti‐Tuj1 (rabbit, 1:200; 5568, Cell Signaling Technology), anti‐Tuj1 (mouse, 1:500; 4466, Cell Signaling Technology), anti‐TRA‐1‐60 (mouse, 1:100; MA1‐023, Thermo Fisher), and anti‐OCT4 (rabbit, 1:100; 11263‐1‐AP, Thermo Fisher). After washing with PBS, sections were incubated for 1 h at room temperature with fluorophore‐conjugated secondary antibodies: Alexa Fluor 488 goat anti‐rat (1:1000; A11006, Invitrogen), Alexa Fluor 488 goat anti‐mouse (1:1000; A21121, Invitrogen), and Alexa Fluor 594 goat anti‐rabbit (1:1000; A11012, Invitrogen). Nuclei were counterstained with Hoechst 33342 (1:10,000; 62249, Thermo Fisher). Finally, sections were mounted with Aquamount (F4680, Sigma‐Aldrich) under cover glasses (Fisher Scientific) and imaged using either a Leica DMi8 fluorescence microscope or a Nikon A1 confocal microscope. Images were processed and analyzed using Fiji (ImageJ). To assess morphological changes of transplanted organoids over time in the cortical and hippocampal regions, the cells from grifted organoid boundaries were manually traced and measured using the “Freehand Selections” tool in Fiji.

### 4.10 MRI Acquisition and Image Analysis

Mice were scanned using a Bruker 9.4 T MRI system. T2‐weighted images were acquired with a spin‐echo sequence (TR/TE = 2500/33 ms) to assess the transplanted organoids at three time points: day ‐3 (7 days post‐stroke), and day 1 and 14 post‐implantation. Diffusion‐weighted imaging (DWI) was performed across the brain using a spin‐echo echo‐planar imaging (EPI) sequence (TR/TE = 2300/25 ms, *b* = 650 s/mm^2^) with 30 diffusion encoding directions, following a previously published protocol [67]. A total of 11 slices (1 mm thickness each slice) were acquired at 14 days post‐transplantation. Apparent diffusion coefficient (ADC) maps were generated to identify the stroke‐affected slice and delineate stroke regions for subsequent region‐of‐interest (ROI) analysis. All other MRI scans were performed using single‐slice acquisitions. Images analysis was conducted in Fiji (ImageJ). Organoid regions were manually delineated using the “Freehand Selections” tool and stored in the ROI Manager. Organoid volume was quantified only at day 1 post‐transplantation using the formula: Volume = area × slice thickness, where area is the sum of segmented ROIs across relevant slices. T2 signal intensity was measured at both days 1 and 14 post‐implantation. For normalization, anatomically matched contralateral regions were manually selected as reference ROIs. The signal intensity ratio between ipsilateral (organoid‐containing) and contralateral regions was calculated to assess temporal changes in graft signal while controlling for inter‐animal variability. DTI‐derived images—including fractional anisotropy (FA), trace, signal intensity, trace‐weighted images, tensor components (Dxx, Dyy, Dzz, Dxy, Dxz, Dyz), eigenvalues (λ1, λ2, λ3), and eigenvectors (X, Y, Z components of the first, second, and third eigenvectors)—were computed directly on the scanner. All datasets were exported using custom MATLAB scripts (MathWorks, Natick, MA, USA) for further quantitative analysis.

### 4.11 BLI of CO Grafts

Graft viability was longitudinally monitored using BLI. Mice were anesthetized and intraperitoneally injected with D‐luciferin (eLUCK‐1 g, Gold biotechnology) at a dose of 150 mg/kg body weight. Images were acquired every 5 min post‐injection until the luminescence signal reached its peak intensity. For quantitative analysis, regions of interest (ROIs) were manually defined over the graft site, and total photon flux (photons/second, P/s) was calculated using Living Image software.

### 4.12 Two‐Photon Imaging and Analysis

For in vitro imaging, live labeled cortical organoids were placed in a shallow indentation formed in a solidified 0.6% agarose layer in a 3.5‐cm dish and kept hydrated with an appropriate volume of culture medium. Two‐photon imaging was performed using a custom‐built two‐photon microscope equipped with a resonant scanner. Fluorophores were excited at selected wavelengths using a femtosecond laser system (Chameleon Discovery, Coherent) focused through an Olympus 25×, 1.05‐NA objective. Emitted fluorescence photons were reflected by a dichroic long‐pass beam splitter (FF705‐Di01–25×36, Semrock), split with a dichroic mirror (565DCXR, Chroma), and detected by photomultiplier tubes (PMTs; H16201P‐40/004, Hamamatsu) after filtering with a 510/84‐nm filter (84‐097, Edmund) for the green channel and two 750SP filters (64‐332, Edmund) for the red channel. Images were acquired using ScanImage (Vidrio Technologies). Three‐dimensional z‐stack images were acquired at a step size of 2 µm along the z‐axis. For multicolor‐labeled organoids, excitation wavelengths were selected based on our previously established optimization strategy, with 780 nm used for TagBFP, 842 nm for iRFP‐682, and 1020 nm for mCherry and GCaMP6s.

For in vivo imaging, mice were maintained on a warm blanket at 37°C under anesthesia during imaging. Each imaging session lasted 45 min to 4 h. Multiple imaging planes were acquired within the same mouse when needed. For longitudinal cell tracking, the same graft region was repeatedly imaged at 30‐min intervals for up to 4 h within a single imaging session, with minor adjustments to the imaging plane made as needed to maintain the region of interest over time. Additional imaging was performed in the same mouse at later post‐transplantation time points, including day 4 and day 14. For GCaMP6s calcium imaging, time‐lapse images were acquired at 960 nm excitation for 30 s. Laser power ranged from 4 to 16.8 mW. After imaging, animals were returned to their home cages for recovery.

Imaging data were processed with custom programs written in Fiji [68]. Raw two‐photon imaging data were imported into ImageJ for the generation of composite images. Three‐dimensional image reconstructions were obtained using the ″3D Viewer. To enhance image contrast and resolution prior to quantitative analysis, we applied 2D deconvolution using the Richardson‐Lucy (RL) algorithm for the image shown in Figure 5d. The point spread function (PSF) was empirically measured from 3D image stacks of sub‐diffraction fluorescent beads and averaged to obtain a representative system PSF. Image stacks were separated into their respective fluorescence channels, and each 2D frame was deconvolved independently using the averaged PSF. Deconvolution was carried out for 20 iterations using the RL algorithm, which is well established in fluorescence microscopy for mitigating spatial blurring caused by diffraction and optical aberrations. The custom analysis code used for the longitudinal cell tracking analysis shown in Supplementary Figure S7 is available on GitHub (https://github.com/liangy10/MIPOT).

### 4.13 Statistical Analysis

Data analysis was performed using a combination of standard functions and custom scripts in MATLAB, Prism 10 (GraphPad Software Inc. US) or PAST [69]. Data were tested for normality using the Shapiro–Wilk test. Parametric tests were applied to data that conformed to a normal distribution, whereas non‐parametric tests were used for data that did not. Normally distributed data are presented as bar graphs showing the mean ± STD; non‐normally distributed data are shown as box plots indicating the median and interquartile range (IQR). Box plots display the median and the 25th–75th percentiles, with whiskers drawn in the Tukey style (±1.5 × IQR; Figure  6e). For comparisons involving two factors, two‐way ANOVA followed by Tukey's multiple comparisons test was performed when data met the assumptions of normality (Figure  4d). For comparisons between two groups, unpaired or paired parametric tests were used for normally distributed data (Figure  4c and Figure  3h, respectively), and the Mann Whitney test was used for unpaired, non‐normally distributed data (Figure  6e). Statistical significance was defined as follows: **p < 0.01 ***p< 0.001 and ****p < 0.0001. Medians, IQRs, means, and SEM. values are reported throughout the text as appropriate.

## Author Contributions

Y.L. conceived the concept and designed the research. J.W. performed iPSC culture, differentiation, characterization, stroke modeling, transplantation, in vivo imaging, histology, and data analysis. J.W. and G.Q. performed MRI scan. C. R. designed the two‐component head fixation system. H.T. and C. C. contributed to BLI and data analysis, stroke modeling and histology. M. J and P.W. contributed to the design and imaging of animal experiments. K.W. and J.X. contributed to MRI analysis. B. Y., M.J.L and T.F. contributed to two‐photon imaging analysis. Y. L. and P.W. supervised research. Y.L. and J.W. wrote the manuscript.

## Conflicts of Interest

The authors declare no conflicts of interest.

## Supporting information




**Supporting File**: advs76267‐sup‐0001‐SuppMat.pdf.

## Data Availability

Design files for the removable 3D‐printed head‐fixation system and data analysis are available at https://github.com/liangy10/MIPOT. All data are available from the Lead Contact, Yajie Liang (Yajie.liang@som.umaryland.edu), upon request.
