# Supplementary material for: Intravital Multimodal Imaging of Human Cortical Organoid Transplantation in a Mouse Model of Chronic Stroke
Source: Adv Sci (Weinh). 2026 Jul 14;13(52):e15913. doi: 10.1002/advs.202515913 (PMC13367743; doi:10.1002/advs.202515913)
Supplement: Supplementary file 1 [file ADVS-13-e15913-s001.pdf]

Intravital Multimodal Imaging of Human Cortical Organoids for Chronic Stroke Treatment in Mice

Jinghui Wang[1], Guanda Qiao[1], Honglin Tan[1], Colleen Russel[1], Baixuan Yang[1], Mengyang Jacky Li[2], Kexin Wang[3,4], Jiadi Xu[4,5], Chengyan Chu[1], Miroslaw Janowski[1], Tian-Ming Fu[2,6], Piotr Walczak[1], Yajie Liang[1,*]

[1.]Department of Diagnostic Radiology and Nuclear Medicine, University of Maryland School of Medicine, Baltimore, MD, USA

[2.]Department of Electrical and Computer Engineering, Princeton University, Princeton, NJ, USA

[3.]Department of Biomedical Engineering, Johns Hopkins University, Baltimore, MD, USA

[4.]F.M. Kirby Research Center for Functional Brain Imaging, Kennedy Krieger Research Institute, Baltimore, MD, USA

[5.]Russell H. Morgan Department of Radiology and Radiological Science, Johns Hopkins University School of Medicine, Baltimore, MD, USA

[6.]Omenn Darling Bioengineering Institute, Princeton University, Princeton, NJ, USA

*Corresponding author: Yajie.liang@som.umaryland.edu

Supplementary Figure 1-9

Supplementary Table 1 and 2

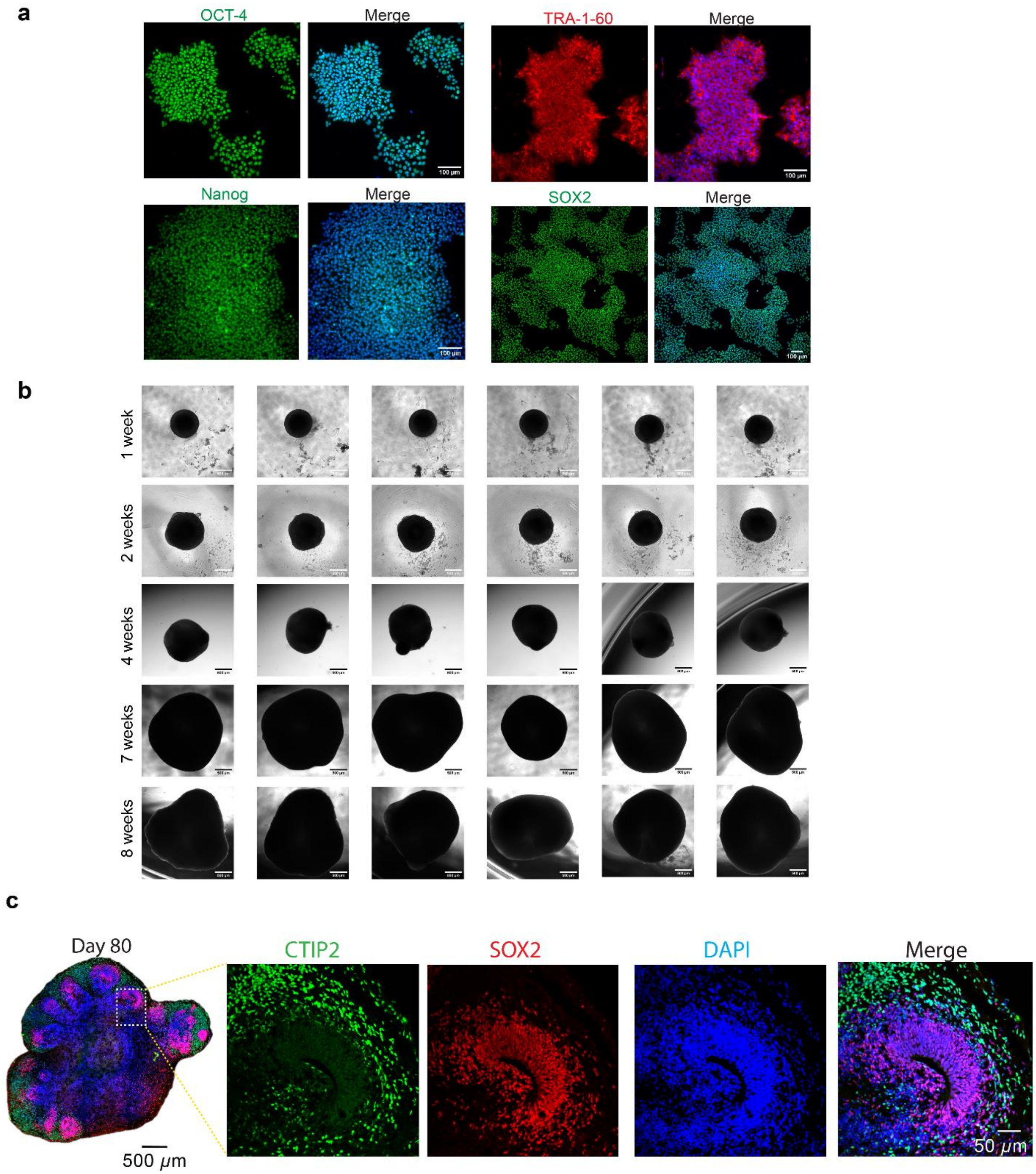

Supplementary Figure 1. Characterization and long-term culture of hiPSC-derived COs.

a. Immunofluorescence staining of pluripotency markers in human induced pluripotent stem cells (hiPSCs). Cells express OCT-4, TRA-1-60, Nanog, and SOX2, confirming maintenance of pluripotency. Nuclei were counterstained with DAPI. Scale bars, 100 μm.

b. Brightfield images of COs at 1, 2, 4, 7, and 8 weeks of differentiation. Organoids exhibit progressive growth and increased opacity over time, indicating tissue maturation. Each time point includes multiple representative organoids to show consistency across samples. Scale bars, 500 μm.

c. Representative image of a D80 cortical organoid stained for CTIP2 (green), SOX2 (red), and DAPI (blue). Right, enlarged views of the boxed region showing the individual channels and merged image.

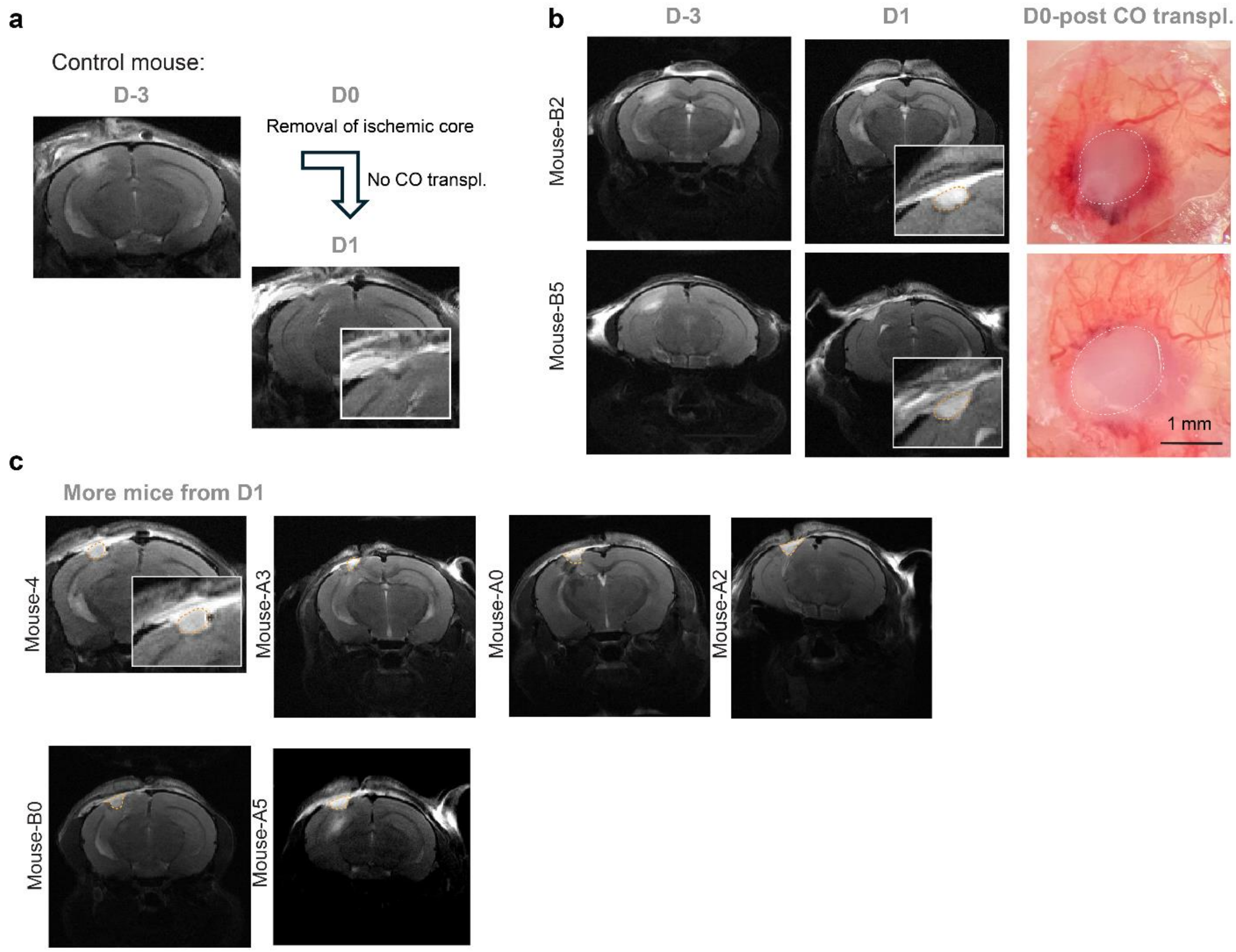

Supplementary Figure 2. Confirmation of CO signal for quantification.

a. T2-weighted MRI of a control mouse imaged by MRI at 3 days before surgery (D-3), D1. The ischemic core was removed on D0, and no COs were transplanted. The inset at D1 shows the resulting cavity, which exhibits a low T2 signal.

b. Representative T2-weighted MRI images of two mice (Mouse-B2 and Mouse-B6) at D-3 and D1 post-transplantation of COs. The insets at D1 show the transplanted COs as distinct T2 hyperintensity regions within the infarct cavity. The right panel displays overhead surgical microscopy photos taken immediately after CO transplantation, confirming the presence of the graft (dashed white outline).

c. T2-weighted MRI images from the most representative planes on Day 1 from all animals (Mouse-4, Mouse-A3, Mouse-A0, Mouse-A2, Mouse-B0, Mouse-A5) included in the study for

quantification in Fig. 3g, demonstrating the successful initial engraftment of COs. The insets show magnified views of the transplanted COs.

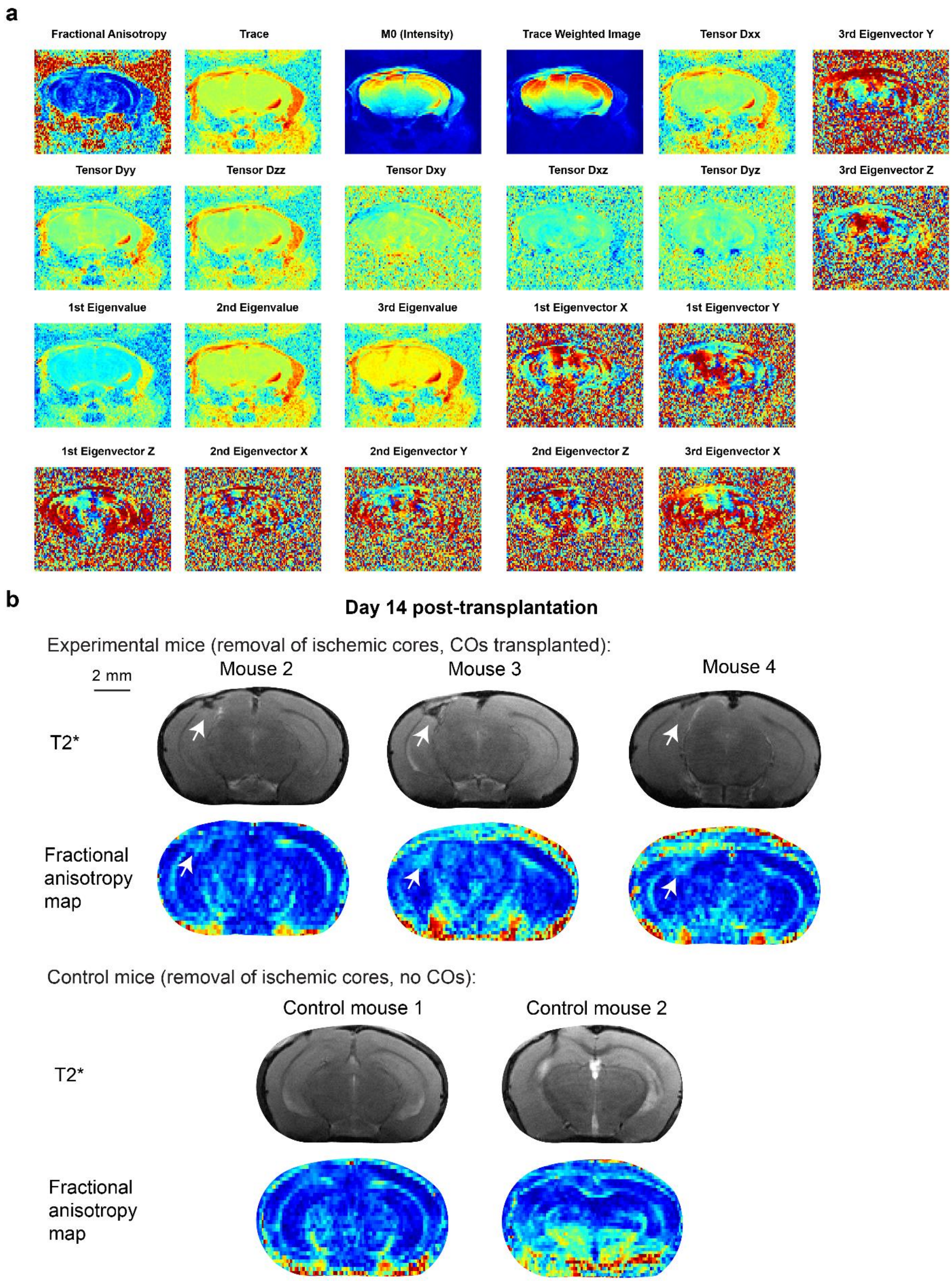

Supplementary Figure 3. Diffusion tensor imaging analysis plots.

a. The 22 parameters plotted from the DTI analysis using jet as the color map.

b. Representative T2*-weighted MRI images and corresponding fractional anisotropy (FA) maps from three additional mice on day 14 post-transplantation. Two control mice were included. Arrows indicate the region of interface-associated FA signal enhancement adjacent to the graft site.

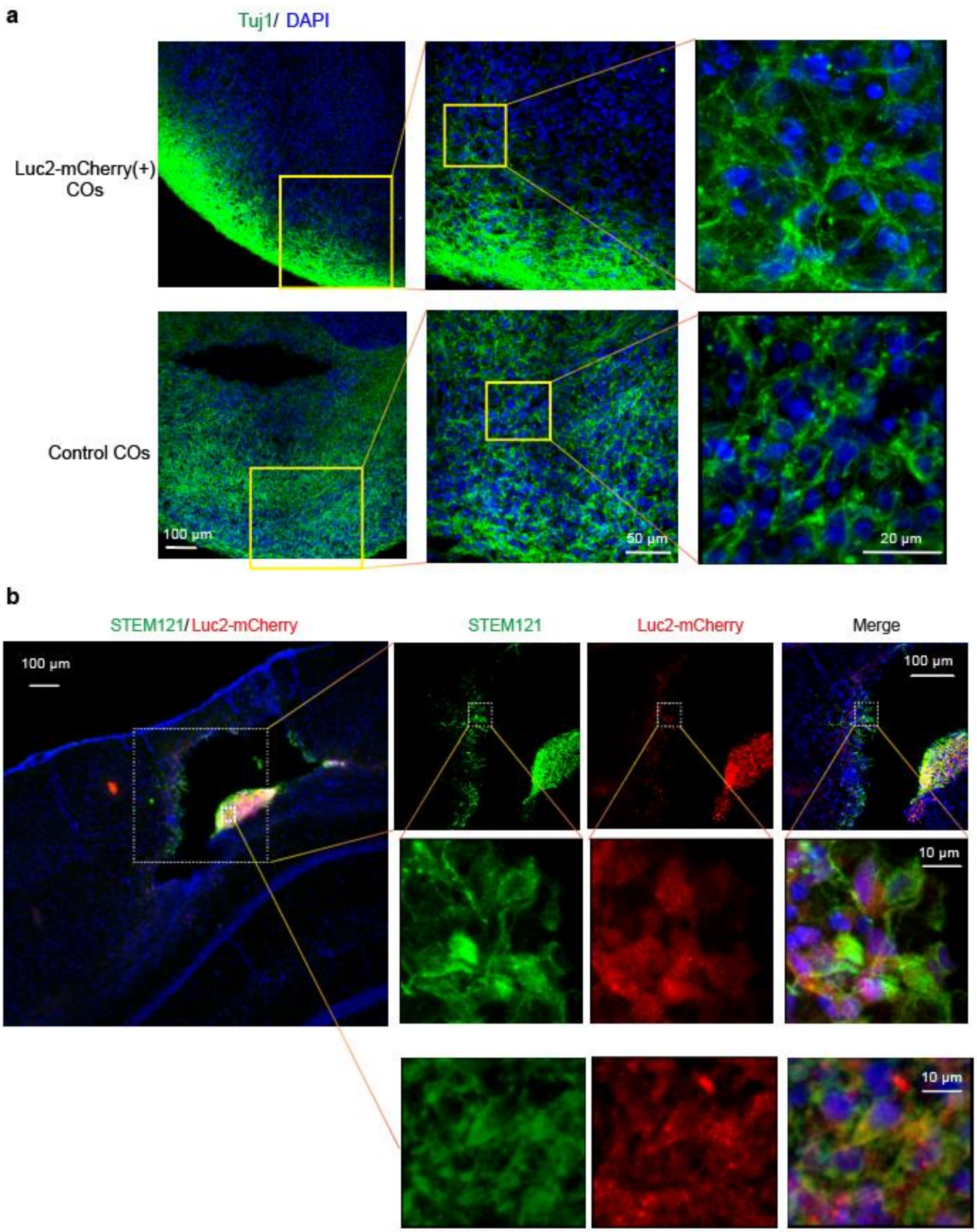

Supplementary Figure 4. Validation of reporter expression and neuronal maturation in Luc2-mCherry–labeled COs.

a. Immunofluorescence staining of COs derived from Luc2-mCherry–labeled hiPSCs (top) and unlabeled controls (bottom), showing neuronal marker Tuj1 (green) and nuclei (DAPI, blue). Representative images at increasing magnifications illustrate comparable neuronal differentiation between groups at the mature stage.

b. Immunostaining of grafted Luc2-mCherry (+) COs 14 days after transplantation into the stroke cavity. Human-specific STEM121 (green) colocalizes with Luc2-mCherry (red), confirming the survival and integration of donor-derived cells. Merged high-magnification images (rightmost panels) show cytoplasmic overlap of both markers.

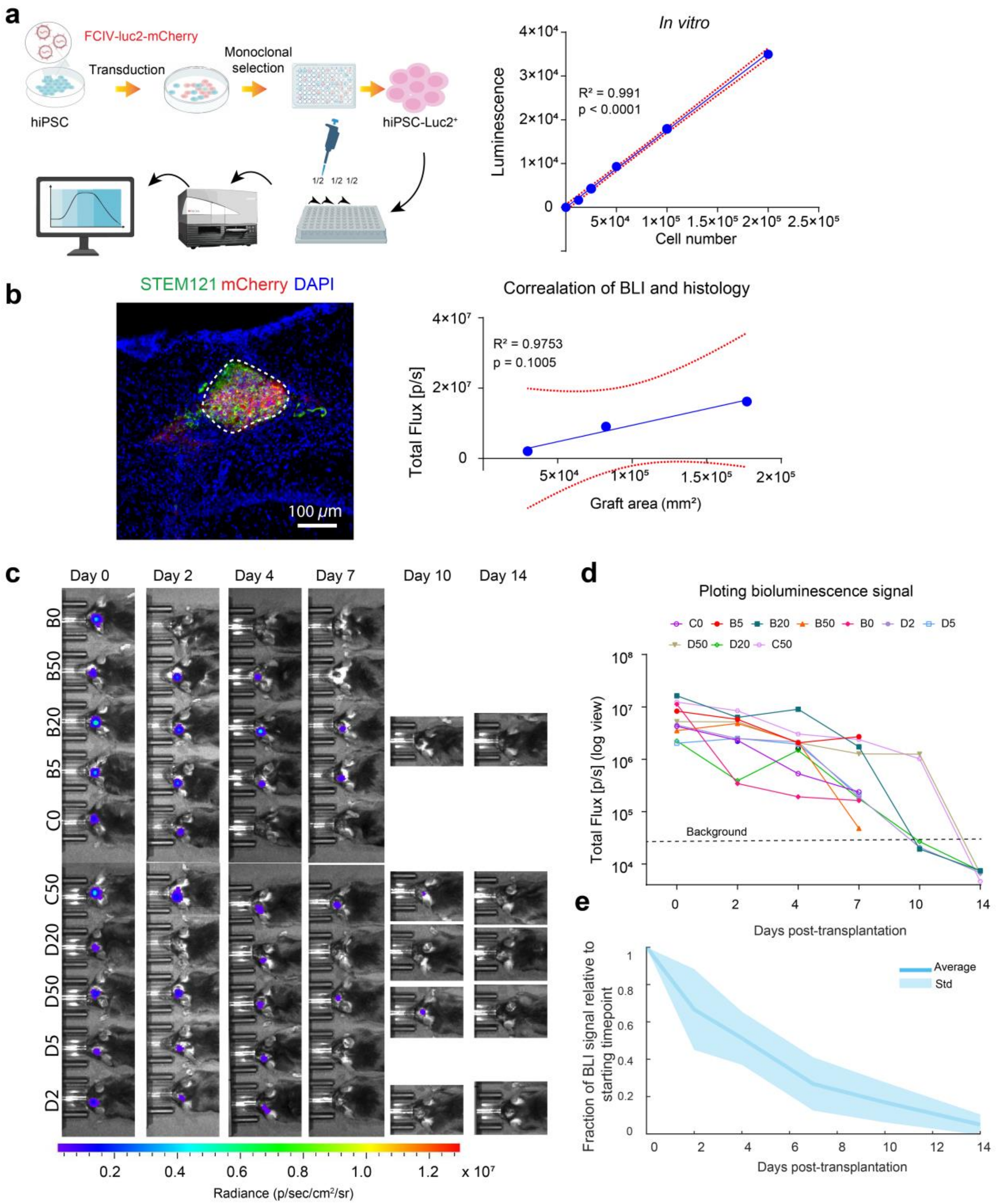

Supplementary Figure 5. Validation of BLI as a readout of graft viability *in vitro* and *in vivo*.

a. Schematic of the in vitro BLI assay workflow. hiPSCs were transduced with the FCIV-luc2-mCherry construct, subjected to monoclonal selection, and serially diluted for BLI measurement in a 96-well plate.

Right, luminescence plotted against cell number, showing a strong linear correlation ($R^2 = 0.991$, $p < 0.0001$).

b. Left, representative immunofluorescence image of a grafted organoid in a brain section stained for STEM121 (green), mCherry (red), and DAPI (blue). The dashed outline indicates the graft area used for histological quantification. Right, correlation between endpoint in vivo BLI signal (total flux) and histologically measured graft area in three animals. A positive trend was observed but did not reach statistical significance ($R^2 = 0.9753$, $p = 0.1005$). Solid blue line, linear regression; dotted red lines, 95% confidence intervals. Scale bar, 100 µm.

c. Serial in vivo BLI images from individual animals acquired at Day 0, 2, 4, 7, 10, and 14 after transplantation (n = 10 mice).

d. Top, longitudinal quantification of total BLI signal (total flux) for each animal shown in c; dashed line indicates background level.

e. Mean BLI signal normalized to the starting timepoint (Day 0 or Day1), shown with shaded standard deviation over time. Summary of all mice (n = 15 mice) including animals shown in (c, n = 10) and Fig. 4 (n = 5).

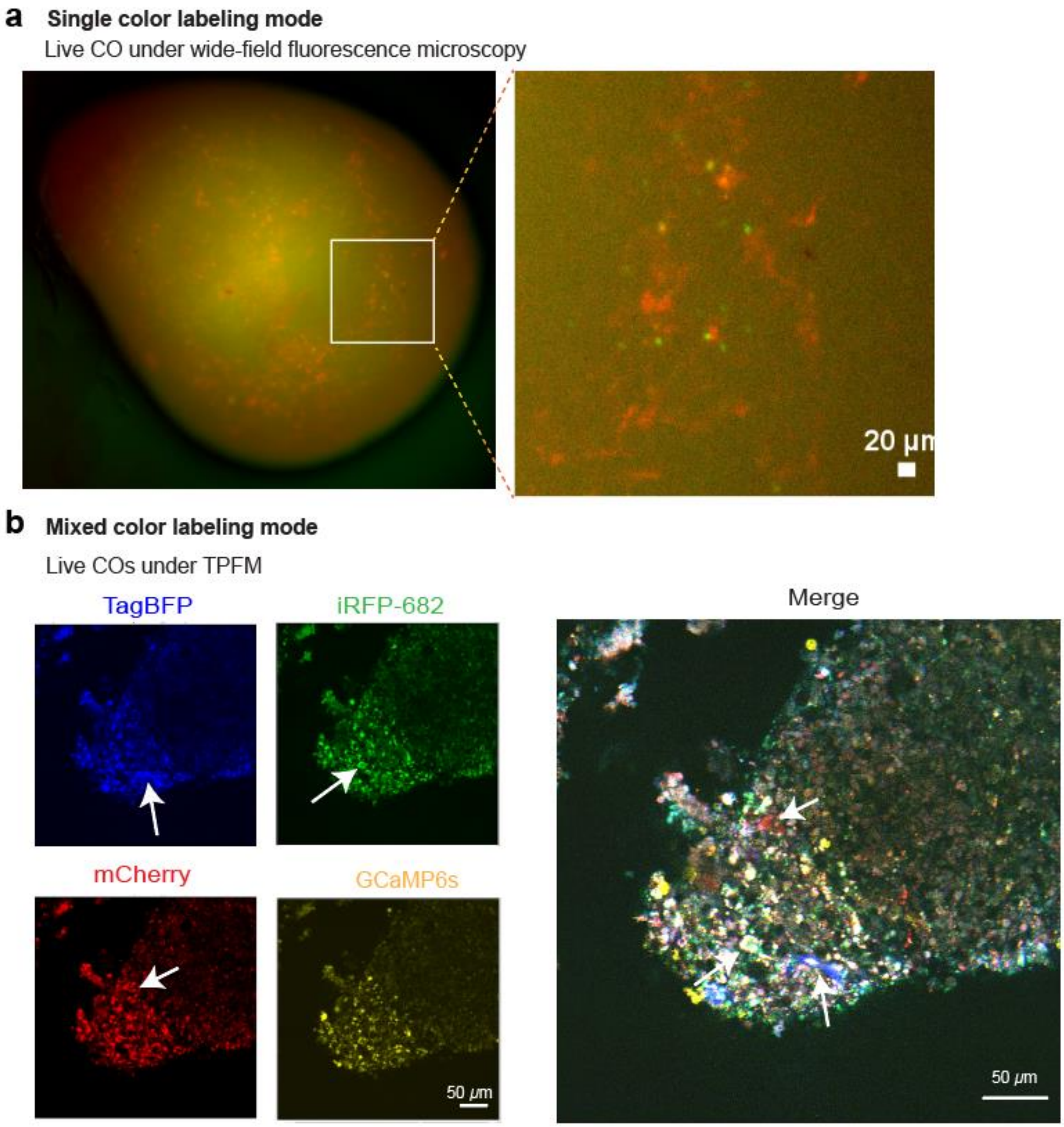

Supplementary Figure 6. Multicolor labeling and high-resolution imaging of live COs using wide-field and two-photon fluorescence microscopy.

a. Representative images of live COs labeled in single-color mode using ICam vectors expressing ICam:mCherry and GCaMP6s in the nucleus. Wide-field fluorescence microscopy revealed distinct mCherry (red) and iRFP-682 (green) expression within the CO, though spatial resolution is limited by tissue

scattering.

b. Mixed-color labeling of live COs using a 1:1:1 ratio of ICam-mCherry, ICam-TagBFP, and ICam-iRFP-682 vectors co-expressing nuclear-localized H2B-GCaMP6s (G6s). TPFM allowed subcellular resolution of individual cells within the CO, each uniquely color-coded by fluorophore combinations (arrows). Multichannel acquisition enabled spectral separation of TagBFP (blue), iRFP-682 (green), mCherry (red), and GCaMP6s (yellow), with the merged image illustrating successful multicolor labeling. Each arrow points to a representative cell. This labeling and imaging approach provides a powerful tool to resolve spatial organization and monitor the behavior and function of individual CO-derived cells.

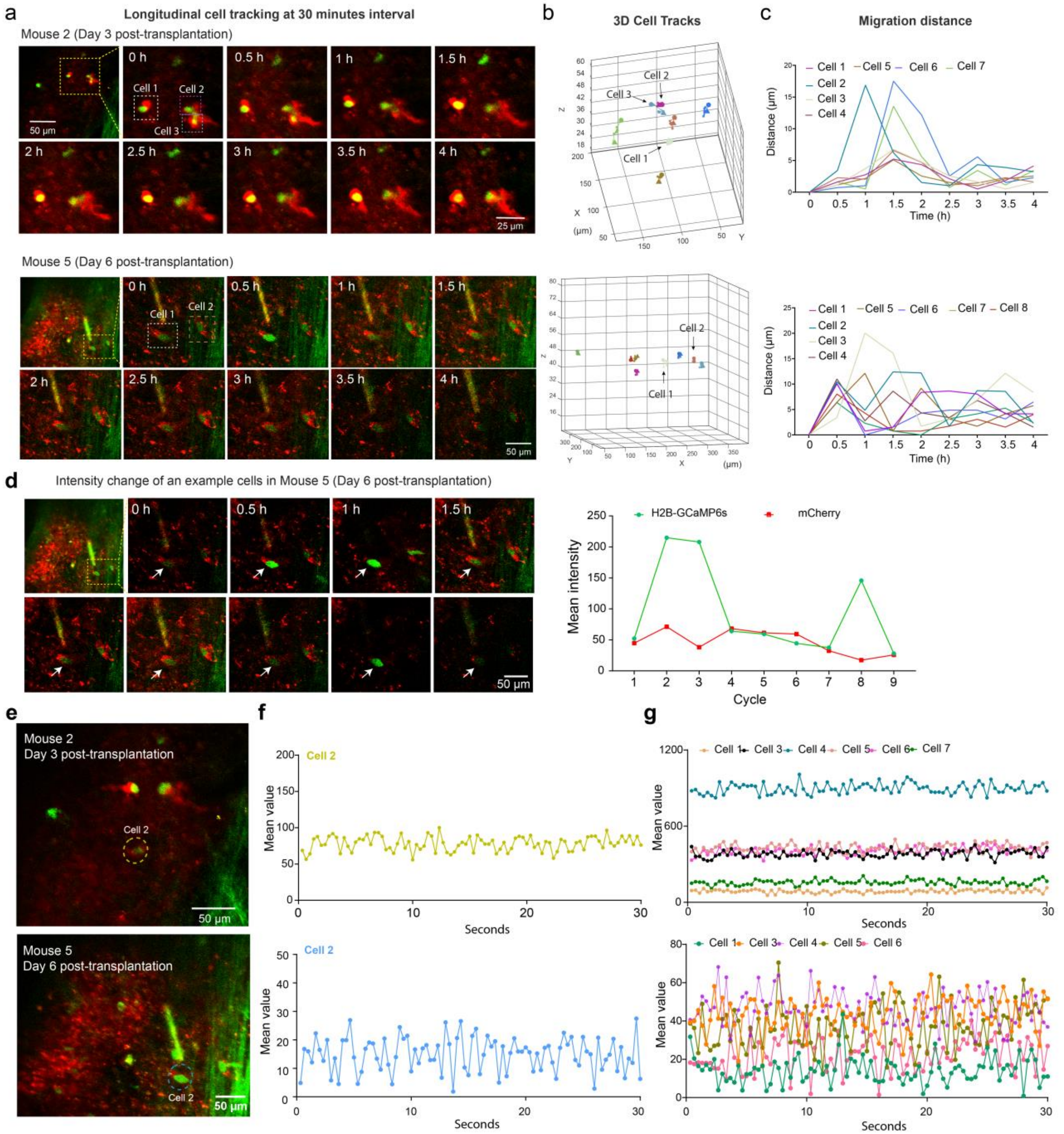

Supplementary Figure 7. In vivo longitudinal cell tracking and timelapse GCaMP6s imaging of grafted CO cells.

a. Representative TPFM images showing longitudinal tracking of grafted cells expressing mCherry and H2B-GCaMP6s at 30 min intervals over 4 h in two mice imaged at different early post-transplantation time points. First row: mouse #2 on day 3 and second row: mouse #5 at day 6. Numbered cells indicate examples tracked across time.

b. Three-dimensional reconstructions of tracked cell trajectories from the cells shown in (**a**).

c. Migration distance over time for individually tracked cells in the two mice.

d. Representative example cell (cell1) from mouse 5 (day 6 post-transplantation) showing changes in signal intensity of a grafted cell during longitudinal imaging. Left, serial TPFM images acquired over 4 h centered on example cell (cell1); arrows indicate the example cell. Right, mean intensity values of H2B-GCaMP6s (green) and mCherry (red) measured from the same cell across imaging cycles.

e. Representative fields used for short-term time-lapse GCaMP6s imaging, with example cells outlined.

f. Representative 30 s fluorescence traces from the example cells shown in (**d**) with a sampling rate of 3Hz excited by 1000 nm two-photon laser.

g. Fluorescence traces from multiple grafted cells recorded over 30 s in each mouse. 1000 nm two-photon lasers were used for excitation in all panels. Imaging in **f,g** was performed at 3 Hz. Scale bars are indicated in each panel.

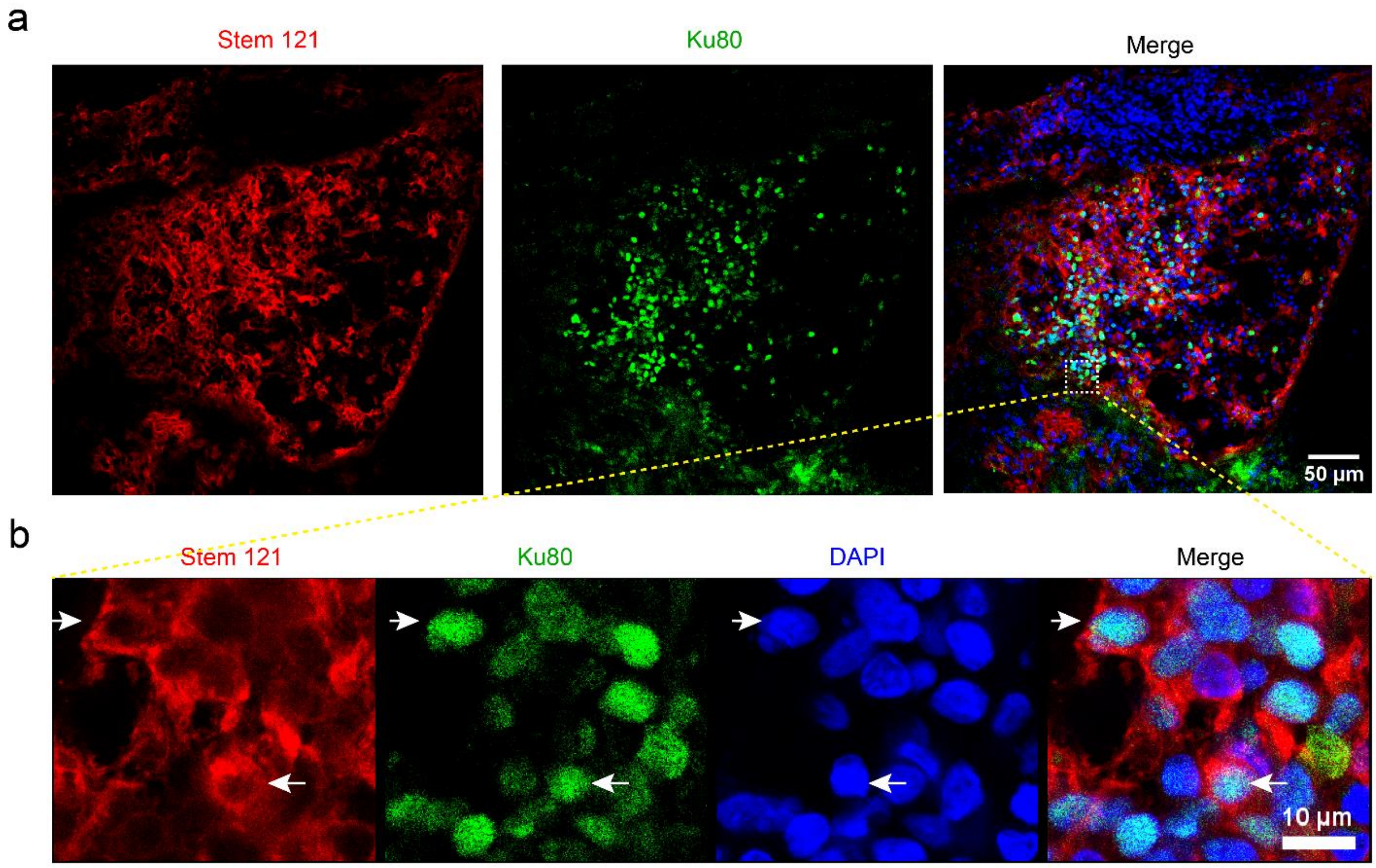

Supplementary Figure 8. Ku80 staining confirms survival of transplanted human organoid cells at 4 weeks post-transplantation.

a. Low-magnification immunofluorescence images of the graft region stained for STEM121 (red), Ku80 (green), and DAPI (blue). Merged image shows Ku80-positive human nuclei within the STEM121-positive graft area.

b. Higher-magnification views of the boxed region in a. Arrows indicate representative Ku80-positive nuclei associated with STEM121-positive cellular structures. Scale bars, 50 µm (a) and 10 µm (b).

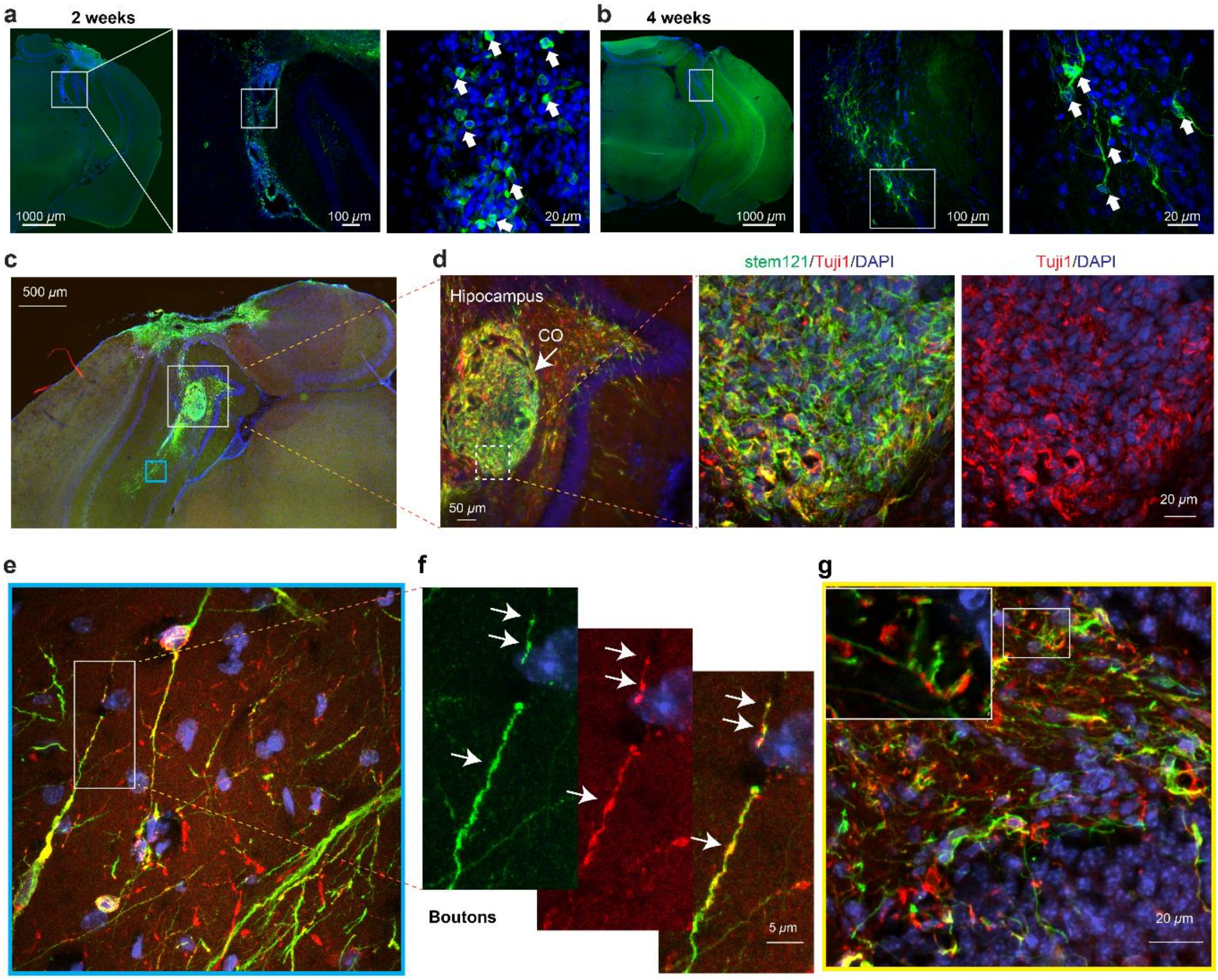

Supplementary Figure 9. Descriptive histological observations of graft-derived cells incidentally detected in hippocampal regions.

a, b Representative images showing STEM121-positive graft-derived human cells at 2 weeks and 4 weeks post-transplantation. White arrows indicate STEM121-positive cells. Scale bars: 1000 μm, 100 μm, and 20 μm as indicated.

c, d Low- and high-magnification images showing graft-derived cells incidentally located in the hippocampal region at 4 weeks post-transplantation. Sections were stained for STEM121, TUJ1, and DAPI. The boxed regions indicate areas shown at higher magnification. Scale bars: 500 μm, 50 μm, and 20 μm as indicated.

e–g Higher-magnification images of hippocampal graft-associated cells showing extended neurite-like morphology and TUJ1 expression. Arrows indicate neurite-like processes or bouton-like structures. Scale bars: 20 μm and 5 μm as indicated.

Supplementary Table 1. Comparison with previous most relevant work

Author / year	Species	Modeling method	Timing (post-stroke)	Graft	In vivo cell tracking	COs were transplanted into	Link
Wang SN et al., 2020 (Translational Stroke Research)	Rat (SD)	MCAO	6 h, 24 h, 7 d	hCOs	No	Biopsy-punched cavity in motor cortex (3 mm Ø, 2 mm depth)	PMID:31889243
Cao SY et al., 2023 (npj Regenerative Medicine)	Mouse (NOD-SCID)	Photothrombosis	7 d	hCOs	No	Junction of infarct core and peri-infarct zone (3 sites surrounding core)	PMID:37253754
Cao SY et al., 2023 (BBRC)	Mouse (NOD-SCID)	Photothrombosis	~7 d	hMGE organoids	No	Junction of infarct core and peri-infarct zone	PMID:37300945
Our work	Mouse	Photothrombosis	10 d	hCOs	Yes	Cavity after removal of the ischemic core	—

Supplementary Table 2. Animal grouping (PT: Post-transplantation;D: Day)

Modality		MRI				BLI								TPFM				Endpoint	Used in Figures
Animal-ID	After stroke	PT-D1	PT-D4	PT-D7	PT-D14	PT-D0	PT-D1	PT-D2	PT-D3	PT-D4	PT-D7	PT-D10	PT-D14	PT-D0	PT-D3	PT-D6	PT-D14		
A-0#	✓	✓			✓													4 weeks	Sup. Fig 2, Sup. Fig 3
A-2#	✓	✓																4 weeks	Sup. Fig 2
A-3#	✓	✓																4 weeks	Fig 7, Sup. Fig 2, Sup Fig 9
A-5#	✓	✓			✓													4 weeks	Sup. Fig 2
Mouse 4	✓	✓																Day 2	Sup. Fig 2
A-20#	✓	✓			✓													4 weeks	Sup. Fig 3
B-0#	✓	✓			✓													2 weeks	Fig 3, Fig 7, Sup. Fig 2, Sup Fig 9
B-2#	✓	✓															✓	2 weeks	Fig 5, Sup. Fig 2
B-3#	✓	✓			✓												✓	4 weeks	Fig7, Sup. Fig 3, Sup Fig 9
B-5#	✓	✓			✓												✓	2 weeks	Sup. Fig 2
0#							✓		✓		✓	✓	✓					2 weeks	Fig 4, Sup. Fig 4, Sup Fig 5
2#							✓		✓		✓	✓	✓					2 weeks	Fig 4
5#							✓		✓		✓	✓	✓					2 weeks	Fig 4

20#							✓		✓		✓		✓					Died at day 10	Fig 4
50#							✓		✓		✓	✓	✓					2 weeks	Fig 4, Sup. Fig 5
Sham -B-2#	✓	✓		✓	✓													2 weeks	Sup. Fig 3
Sham -C-5#	✓	✓		✓	✓													2 weeks	Sup. Fig 3
B-5#	✓	✓		✓		✓		✓		✓	✓							Day 7	Sup. Fig 5
C-0#	✓	✓		✓		✓		✓		✓	✓							Day 7	Sup Fig 5
D-5#	✓	✓		✓				✓		✓	✓							Day 7	Sup. Fig 5
B-20#	✓	✓		✓		✓		✓		✓	✓	✓	✓					2 weeks	Sup. Fig 5
C-50#	✓	✓		✓		✓		✓		✓	✓	✓	✓					2 weeks	Sup. Fig 5
D-2#	✓	✓		✓		✓		✓		✓	✓	✓	✓					2 weeks	Sup. Fig 5
D-20#	✓	✓		✓		✓		✓		✓	✓	✓	✓					2 weeks	Sup. Fig 5
D-50#	✓	✓		✓		✓		✓		✓	✓	✓	✓					2 weeks	Sup. Fig 5
A-2#-window	✓		✓	✓	✓	✓		✓		✓	✓	✓	✓		✓		✓	2 weeks	Fig 6, Sup. Fig 7, Sup. Fig 5
A-5#-window	✓		✓	✓		✓		✓		✓	✓	✓	✓	✓		✓		Died at day 10	Sup. Fig 7
B-0#	✓	✓				✓		✓		✓	✓							Died at day 7	Sup. Fig 5
B-50#	✓	✓				✓		✓		✓	✓							Died at day 7	Sup. Fig 5

255
